# *Pseudomonas aeruginosa* Biofilm Dispersion by the Human Atrial Natriuretic Peptide

**DOI:** 10.1002/advs.202103262

**Published:** 2022-01-14

**Authors:** Mélissande Louis, Thomas Clamens, Ali Tahrioui, Florie Desriac, Sophie Rodrigues, Thibaut Rosay, Nicholas Harmer, Suraya Diaz, Magalie Barreau, Pierre‐Jean Racine, Eric Kipnis, Teddy Grandjean, Julien Vieillard, Emeline Bouffartigues, Pierre Cornelis, Sylvie Chevalier, Marc G. J. Feuilloley, Olivier Lesouhaitier

**Affiliations:** ^1^ Laboratory of Microbiology Signals and Microenvironment LMSM EA 4312 University of Rouen Normandy Evreux 27000 France; ^2^ Normandie Univ UNICAEN Unité De Recherche Risques Microbiens U2RM Caen 14000 France; ^3^ School of Biosciences University of Exeter Exeter EX4 4QD UK; ^4^ Univ. Lille CNRS Inserm, CHU Lille Institut Pasteur de Lille U1019‐UMR9017‐CIIL‐Centre d’Infection et d’Immunité de Lille, Lille, France University Lille Lille F‐59000 France; ^5^ Normandie Univ UNIROUEN INSA Rouen CNRS COBRA (UMR 6014) Evreux 27000 France

**Keywords:** *ami* pathway, antibiotics, bacterial adaptation, bacterial sensor, biofilm dispersion, hANP, natriuretic peptides, *Pseudomonas aeruginosa*

## Abstract

*Pseudomonas aeruginosa* biofilms cause chronic, antibiotic tolerant infections in wounds and lungs. Numerous recent studies demonstrate that bacteria can detect human communication compounds through specific sensor/receptor tools that modulate bacterial physiology. Consequently, interfering with these mechanisms offers an exciting opportunity to directly affect the infection process. It is shown that the human hormone Atrial Natriuretic Peptide (hANP) both prevents the formation of *P. aeruginosa* biofilms and strongly disperses established *P. aeruginosa* biofilms. This hANP action is dose‐dependent with a strong effect at low nanomolar concentrations and takes effect in 30–120 min. Furthermore, although hANP has no antimicrobial effect, it acts as an antibiotic adjuvant. hANP enhances the antibiofilm action of antibiotics with diverse modes of action, allowing almost full biofilm eradication. The hANP effect requires the presence of the *P. aeruginosa* sensor AmiC and the AmiR antiterminator regulator, indicating a specific mode of action. These data establish the activation of the *ami* pathway as a potential mechanism for *P. aeruginosa* biofilm dispersion. hANP appears to be devoid of toxicity, does not enhance bacterial pathogenicity, and acts synergistically with antibiotics. These data show that hANP is a promising powerful antibiofilm weapon against established *P. aeruginosa* biofilms in chronic infections.

## Graphical abstract

This work presents evidence that the human Atrial Natriuretic Peptide (hANP) hormone can represent a powerful agent to efficiently disperse established biofilms of *Pseudomonas aeruginosa* even at extremely low concentrations. hANP has high therapeutic potential on its own (no toxicity). It also acts as an adjuvant, enhancing different antibiotics’ effects on *P. aeruginosa* by almost fully eradicating established biofilms.

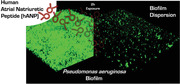

## 1 Introduction

Many bacteria switch from a planktonic state to a sessile lifestyle during infection, and this is critical to their virulence and resistance to antibiotic therapy. In host tissues, bacteria that establish in a biofilm present a high tolerance to antimicrobials and the host immune defense.^[^ ^1^, ^2^ ^]^ In addition, a small percentage of biofilm persister cells becomes highly tolerant to antibiotics favoring the relapse of infections.^[^ ^3^, ^4^, ^5^, ^6^ ^]^ Treating such infections requires the use of high and repeated doses of antibiotics, which is associated with deleterious effects in treated patients.^[^ ^7^, ^8^ ^]^ The well‐known opportunistic pathogen *Pseudomonas aeruginosa* is a major cause of mortality in cystic fibrosis (CF)‐suffering individuals.^[^ ^9^, ^10^ ^]^ *P. aeruginosa* also causes medical device‐related infections, as well as wounds and keratitis infections due to its ability to readily form biofilms.^[^ ^11^, ^12^ ^]^ Furthermore, *P. aeruginosa* has been classified in the ESKAPE group of bacteria (*Enterococcus faecium*, *Staphylococcus aureus*, *Klebsiella pneumoniae*, *Acinetobacter baumannii*, *Pseudomonas aeruginosa*, and *Enterobacter spp*).^[^ ^13^ ^]^ These represent the most critical bacteria in terms of both antibiotic resistance and the ability to form recalcitrant biofilms urgently needing new therapeutic drugs.^[^ ^14^, ^15^, ^16^, ^17^ ^]^

In this global context, numerous nonantibiotic antivirulence agents such as quorum‐sensing inhibitors, enzymes, or peptides have been proposed as alternative antimicrobial weapons^[^ ^18^, ^19^, ^20^ ^]^ and/or as biofilm dispersal agents.^[^ ^21^, ^22^, ^23^, ^24^, ^25^, ^26^ ^]^ At least two main avenues are being explored to tackle biofilms. The first is the search for new bactericidal agents.^[^ ^4^ ^]^ However, biofilm eradication usually requires the use of high doses of antimicrobials that poses a high risk for the emergence of resistance.^[^ ^4^ ^]^ The second strategy focuses on the development of natural or synthetic agents able to disperse established biofilms with low or no effect on bacterial viability.^[^ ^25^ ^]^ These would promote both the clearance of the biofilm and the action of antibacterial agents that are more active on planktonic cells. The endogenous peptides produced by cells involved in human innate immunity, as well as small synthetic peptides displaying broad‐spectrum antibiofilm activity, have been proposed to eradicate pan‐antibiotic‐resistant bacteria.^[^ ^27^, ^28^ ^]^ These host molecules provide a promising start point for the development of new antibacterial and antibiofilm agents.^[^ ^29^, ^30^ ^]^ These peptides have been tested either alone^[^ ^31^ ^]^ or in combination with antibiotics.^[^ ^32^ ^]^ However, some of them (e.g., cathelicidin LL‐37 and magainin II cationic antimicrobial peptides) also target bacterial membranes, which again risks the emergence of bacterial resistance.^[^ ^33^ ^]^

Host communication molecules have recently emerged as an interesting alternative in the search for compounds that modulate bacterial virulence or biofilms rather than killing bacteria. The impact of eukaryotic signaling compounds on bacteria represents an interesting and emerging field of research termed “*Microbial Endocrinology*.''^[^ ^34^ ^]^ Cytokines, neurotransmitters, and hormones can modulate bacterial physiology.^[^ ^35^, ^36^, ^37^, ^38^ ^]^ *P. aeruginosa* responds to some of these eukaryotic communication molecules,^[^ ^39^ ^]^ such as the interferon‐*γ* cytokine^[^ ^40^ ^]^ or the dynorphin peptide^[^ ^41^ ^]^ at least partly through their specific binding to bacterial sensors.^[^ ^37^, ^40^, ^41^ ^]^

The most investigated hormone peptides active on bacteria are the natriuretic peptides (NP) family. This family is composed of three main members, the atrial (ANP), the brain (BNP), and the c‐type natriuretic peptide (CNP).^[^ ^42^ ^]^ The acetamide binding AmiC protein has been identified as the *P. aeruginosa* NP sensor.^[^ ^43^, ^44^ ^]^ Notably, AmiC can discriminate between different NPs,^[^ ^44^ ^]^ as is the case for the human NP subtype C (hNPR‐C),^[^ ^42^, ^45^ ^]^ which therefore makes AmiC an hNPR‐C receptor equivalent. Recent studies have shown that while both hCNP and human BNP (hBNP) strongly prevent *P. aeruginosa* biofilm formation, the two peptides act through distinct mechanisms.^[^ ^43^, ^44^ ^]^ hCNP was shown to act directly on AmiC at low concentrations (0.1 × 10^−6^ m or less), while inducing a general stress response at higher doses (1 × 10^−6^ m or more).^[^ ^43^, ^44^ ^]^ On the other hand, hBNP effects seem to be relayed by an unidentified sensor different from AmiC or by a nonspecific mechanism.^[^ ^44^ ^]^ These data suggest that bacterial responses differ depending on the NPs and their concentrations.

The aim of the present study is therefore to investigate the impact of the third NP, ANP, on *P. aeruginosa* biofilm formation as well as its dispersion. Here, we show that hANP not only inhibits biofilm formation but also strongly disperses established biofilms, even at very low concentrations. This dispersal activity appears to be hANP‐specific and is relayed by the AmiC sensor and its associated AmiR regulator protein. Furthermore, hANP could act as an adjuvant potentiating the effect of common antibiotics used to treat *P. aeruginosa* infections resulting in almost fully eradication of *P. aeruginosa* biofilms.

## 2 Results

### 2.1 hANP Hinders *P. aeruginosa* Biofilm Establishment

**Figure 1:**
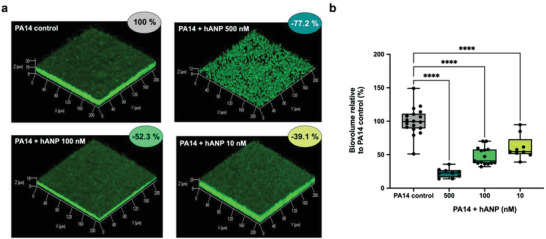
Effect of hANP on *P. aeruginosa* biofilm formation. a) 3D shadow representations of *P. aeruginosa* PA14 biofilm structures, unexposed (control condition) or exposed to hANP (500 × 10^−9^, 100 × 10^−9^, or 10 × 10^−9^ m), grown in dynamic conditions at 37 °C for 24 h. Bacterial cells within biofilms were stained with SYTO9 and observed by CLSM. b) COMSTAT image analyses of biofilms structures of *P. aeruginosa* PA14 exposed during biofilm formation to hANP (500 × 10^−9^, 100 × 10^−9^, or 10 × 10^−9^ m) in dynamic conditions for 24 h at 37 °C, compared to unexposed condition. Data are the result of the analysis of 18 (control condition), nine (hANP 500 × 10^−9^ m), 15 (hANP 100 × 10^−9^ m), and nine (hANP 10 × 10^−9^ m) views from at least three independent biological experiments (*n* = 3). Statistics were achieved by one‐way ANOVA followed by Dunnett's multiple‐comparison test. Values that are significantly different are indicated by asterisks as follows: ****, *p* < 0.0001.

To assess the effect of hANP on *P. aeruginosa* PA14 strain biofilm formation, a dynamic flow‐cell system coupled with confocal laser scanning microscopy (CLSM) was used, in which bacteria were exposed to hANP over 24 h. When PA14 was exposed to 500 × 10^−9^ m hANP, the bacterial biofilm formation was strongly impaired (−77.2 ± 2.3%; *p* < 0.0001) compared to the control condition (**Figure** **1**a,b). This effect appears to be dependent on the hANP concentration in contact with bacteria (Figure 1a). Indeed, exposure of *P. aeruginosa* to a concentration of hANP at 100 × 10^−9^ and 10 × 10^−9^ m resulted in a decrease of bacterial biomass which constitutes the biofilm as compared to the control condition (Figure 1b), with a reduction of 52.3 ± 3.3% and 39.1 ± 5.9%, respectively. These results prompted us to ascertain the impact of hANP on already established biofilms.

### 2.2 hANP Disperses Established *P. aeruginosa* Biofilms at Extremely Low Concentrations

**Figure 2:**
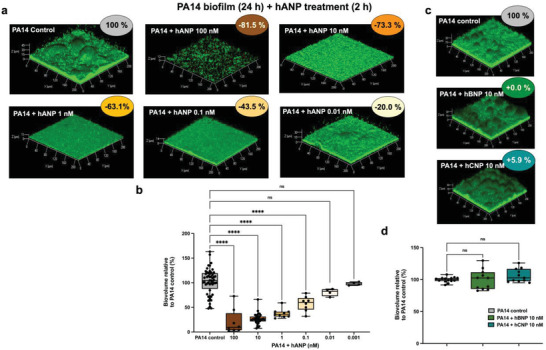
Effect of hANP, Brain Natriuretic Peptide (hBNP), and C‐type Natriuretic Peptide (hCNP) on established biofilm of *P. aeruginosa*. a) 3D shadow representations of 24 h preformed *P. aeruginosa* PA14 biofilm structures exposed to various concentration of hANP (100 × 10^−9^ to 0.01 × 10^−9^ m) for 2 h at 37 °C compared to untreated biofilms (control condition). b) COMSTAT image analyses of biofilms structures of *P. aeruginosa* PA14 control or exposed for 2 h to hANP (100 × 10^−9^, 10 × 10^−9^, 1 × 10^−9^, 0.1 × 10^−9^, 0.01 × 10^−9^, or 0.001 × 10^−9^ m), at 37 °C. Data are the result of the analysis of 30 views from eight independent biological experiments (hANP 100 × 10^−9^ m), 127 measurements from 40 independent experiments (*n* = 40) (hANP 10 × 10^−9^ m), 28 measurements from nine independent experiments (*n* = 9) (hANP 1 × 10^−9^ m), 27 measurement from eight independent experiments (*n* = 8) (hANP 0.1 × 10^−9^ m), 13 measurement from four independent experiments (*n* = 4) (hANP 0.01 × 10^−9^ m), and 13 measurement from four independent experiments (*n* = 4) (hANP 0.001 × 10^−9^ m). c) 3D shadow representations of 24 h preformed *P. aeruginosa* PA14 biofilm structures untreated (control condition) or exposed to hBNP (10 × 10^−9^ m) or hCNP (10 × 10^−9^ m) for 2 h at 37 °C. d) COMSTAT image analyses of biofilms structures of *P. aeruginosa* PA14 control or exposed to hBNP (10 × 10^−9^ m) or hCNP (10 × 10^−9^ m) for 2 h at 37 °C. Data are the result of the analysis of at least 11 views from at least three independent biological experiments (*n* = 3). All biofilms were stained with the SYTO9 and observed by CLSM. Statistics were achieved by ordinary one‐way ANOVA followed by Dunnett's multiple‐comparison test. Values that are significantly different are indicated by asterisks as follows: ***, *p* < 0.001; ****, *p* < 0.0001.

To evaluate the potential of hANP as a biofilm dispersal agent, a *P. aeruginosa* PA14 biofilm was established over 24 h in dynamic conditions to obtain a mature biofilm (**Figure** **2**a). Then, the formed biofilms were exposed to hANP at various concentrations or to ultra‐pure distilled water (control hANP solvent) over 2 h. Under these conditions, hANP strongly affected biofilms, with the complete disruption of the 3D mushroom‐like structures at all tested concentrations (Figure 2a). COMSTAT image analyses indicated that hANP exposure (100 × 10^−9^ m) caused a biofilm biovolume reduction of 81.5 ± 4.1% (*p* < 0.0001) when compared to control (Figure 2b). Moreover, dispersion of the preformed biofilm was strongly dependent on the hANP concentration (Figure 2a). hANP reduced *P. aeruginosa* biofilms at a concentration as low as 0.01 × 10^−9^ m (−20.0 ± 3.5%) while hANP at 0.1 × 10^−9^ m dispersed nearly half of the 24 h old established biofilm (−43.5 ± 4.0%; *p* < 0.001) as compared to the control (Figure 2b). Remarkably, hANP at both 1 × 10^−9^ and 10 × 10^−9^ m concentrations reduced the preformed biofilm by 63.1 ± 2.2% (*p* < 0.0001) and 73.3 ± 1.2% (*p* < 0.0001), respectively. In addition, 30 min exposure of 0.1 × 10^−9^ to 1 × 10^−9^ m hANP was sufficient to cause more than 60% dispersion of a 24 h established biofilm (*p* < 0.0001) (Figure S1a,b, Supporting Information), albeit with a slightly lower efficacy compared to a 2 h hANP treatment. Based on these data, 10 × 10^−9^ m hANP was selected as the reference dose for all subsequent experiments.

To ascertain if the biofilm dispersal effect is hANP‐specific compared to other human NPs, the impact of hBNP and hCNP on PA14 preformed biofilms was evaluated. Contrary to hANP, 10 × 10^−9^ m of either hBNP or hCNP did not disrupt 24 h established *P. aeruginosa* PA14 biofilms (Figure 2c,d), suggesting that the dispersion effect is indeed specific to hANP.

To assess whether hANP exerts a biofilm dispersal activity in other *P. aeruginosa* strains, similar assays were performed using PAK and H103 (a prototroph of the wild‐type PAO1) strains belonging to phylogenetic groups of *P. aeruginosa* isolates other than the PA14 group.^[^ ^46^, ^47^ ^]^ 24 h biofilms grown in dynamic conditions were exposed to 10 × 10^−9^ m hANP for 2 h. CLSM observations demonstrated that hANP also substantially dispersed the biofilms of PAK (Figure S2a,b, Supporting Information) and H103 (Figure S2c,d, Supporting Information) strains. The hANP‐dispersal effect toward H103 and PAK biofilms appears slightly lower compared to PA14. This discrepancy in the biofilm sensitivity to hANP exposure of different *P. aeruginosa* strains prompted us to assess the impact of hANP on a panel of eight clinical strains isolated from patients from different French hospitals. We observed substantial heterogeneity in the clinical isolates’ response to hANP (Figure S3, Supporting Information). Interestingly, the strain MUC‐P4 appears to be significantly highly sensitive to 10 × 10^−9^ m hANP (92% biofilm dispersion), MUC‐P5 isolate is weakly sensitive (15% to 30%), and CF 8.19, PAL 0.1, and PAL 1.1 isolates are sensitive in the same range as the H103 and PAK lab strains (50% to 60%). Strikingly, three clinical isolates (CF 9.19, MUC‐N1, and MUC‐N2) are insensitive to hANP treatment.

Overall, these data indicate that hANP, even at low concentrations, strongly disperses established biofilms of *P. aeruginosa* PA14 in a dose‐dependent manner. Interestingly, the biofilm of PA14 appears to be unaffected when challenged by the two other tested NPs (hBNP and hCNP) revealing a specific hANP dispersal effect. Furthermore, hANP also disperses preformed biofilms of other *P. aeruginosa* strains, including clinical isolates, although presenting a highly variable activity ranging from no effect to effects close to total dispersion of the biofilm.

### 2.3 hANP Potentiates Antibiotics for the Eradication of *P. aeruginosa* Biofilm

**Figure 3:**
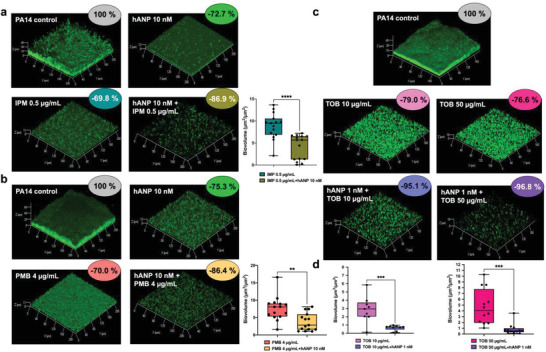
Synergistic effect of hANP in combination with antibiotics on established biofilm of *P. aeruginosa*. a) 3D shadow representations of 24 h old *P. aeruginosa* PA14 biofilm structures untreated (control condition) or treated with hANP (10 × 10^−9^ m), imipenem (IPM) (0.5 µg mL^−1^), or the combination of IPM (0.5 µg mL^−1^) and hANP (10 × 10^−9^ m). COMSTAT image analyses of 24 h old biofilm structures of *P. aeruginosa* PA14 exposed to IPM (0.5 µg mL^−1^) or a combination of IMP (0.5 µg mL^−1^) and hANP (10 × 10^−9^ m). Data are the result of the analysis of 15 views from five independent biological experiments (*n* = 5). Statistics were achieved by a two‐tailed *t* test. Asterisks indicate values that are significantly different as follows: ****, *p* < 0.0001. b) 3D shadow representations of 24 h old *P. aeruginosa* PA14 biofilm structures untreated (control condition) or treated with hANP (10 × 10^−9^ m), polymyxin B (PMB) (4 µg mL^−1^), or the combination of PMB (4 µg mL^−1^) and hANP (10 × 10^−9^ m). COMSTAT image analyses of 24 h old biofilm structures of *P. aeruginosa* PA14 exposed to PMB (4 µg mL^−1^) or a combination of PMB (4 µg mL^−1^) and hANP (10 × 10^−9^ m). Data are the result of the analysis of 15 views from five independent biological experiments (*n* = 5). Statistics were achieved by a two‐tailed *t* test. Asterisks indicate values that are significantly different as follows: **, *p* < 0.01. c) 3D shadow representations of 24 h preformed *P. aeruginosa* PA14 biofilm structures in control condition (untreated) or exposed to tobramycin (TOB) at 10 or 50 µg mL^−1^, or to a combination of TOB (at 10 or 50 µg mL^−1^) and hANP (1 × 10^−9^ m) for 2 h at 37 °C. d) COMSTAT image analyses of 24 h old biofilm structures of *P. aeruginosa* PA14 exposed to tobramycin at 10 µg mL^−1^ or a combination of tobramycin (10 µg mL^−1^) and hANP (1 × 10^−9^ m) (left panel) and 50 µg mL^−1^ or a combination of tobramycin (50 µg mL^−1^) and hANP (1 × 10^−9^ m) (right panel), for 2 h at 37 °C. Data are the result of the analysis of nine views from three independent biological experiments (*n* = 3) for TOB at 10 µg mL^−1^ and 12 views from four independent biological experiments (*n* = 4) for TOB at 50 µg mL^−1^. Statistics were achieved by a two‐tailed *t* test. Asterisks indicate values that are significantly different as follows: ***, *p* < 0.001.

To evaluate the use of hANP as an adjuvant, a 24 h old *P. aeruginosa* PA14 biofilm was exposed to hANP, different antibiotics commonly used to treat *P. aeruginosa* infections (imipenem, polymyxin B, tobramycin), or to a combination of hANP and antibiotics (**Figure** **3**). Exposure to hANP (10 × 10^−9^ m) alone for 2 h caused a 72.7 ± 2.3% biofilm reduction, while a single imipenem treatment (0.5 µg mL^−1^, 2 h) had a similar effect (69.8 ± 3.0% reduction) (Figure 3a). Exposure to hANP (10 × 10^−9^ m) and imipenem (0.5 µg mL^−1^) combination treatment resulted in a much higher impact on the established biofilm with a significant biovolume reduction of 86.9 ± 3% (Figure 3a). The comparison of the antibiofilm activity of imipenem alone and in combination with hANP was significantly different (*p* < 0.0001). Similarly, combined treatment of hANP (10 × 10^−9^ m) and polymyxin B (4 µg mL^−1^) enhanced biofilm destruction compared to the action of the antibiotic alone (Figure 3b). A single hANP exposure (10 × 10^−9^ m) reduced the biovolume by 75.3 ± 1.7%, polymyxin B (2 h; 4 µg mL^−1^) by 70.0 ± 4.3% (Figure 3b), and the combination of hANP and polymyxin B by 86.4 ± 2.7% (Figure 3b). The comparison of the antibiofilm activity of polymyxin B alone and in combination with hANP was also significantly different (*p* < 0.01). Finally, the impact of a cocktail containing hANP at 1 × 10^−9^ m and tobramycin at two different concentrations (10 and 50 µg mL^−1^) was also assessed (Figure 3c). Interestingly, we observed that tobramycin at 10 and 50 µg mL^−1^ led to a biofilm biovolumes reduction of 79.0 ± 4.0% and 76.6 ± 4.0%, respectively (Figure 3c,d). Addition of hANP at 1 × 10^−9^ m strongly and significantly enhanced the dispersion of the established biofilm in presence of tobramycin. Indeed, the combination between hANP (1 × 10^−9^ m) and tobramycin (10 µg mL^−1^), or tobramycin (50 µg mL^−1^) provoked a massive eradication of the biofilm reaching an ultimate decrease of 95.1 ± 0.7% (*p* < 0.01) or 96.8 ± 0.5% (*p* < 0.001), respectively, as compared to the control condition (Figure 3c,d). Together, our data indicate that hANP, at extremely low concentrations, in combination with antibiotics significantly enhances the eradication of established biofilm of *P. aeruginosa* PA14. Therefore, hANP can function as an adjuvant agent against *P. aeruginosa* chronic infections.

### 2.4 hANP is not a Bactericidal Agent and Alters Biofilm Matrix Polysaccharides

**Figure 4:**
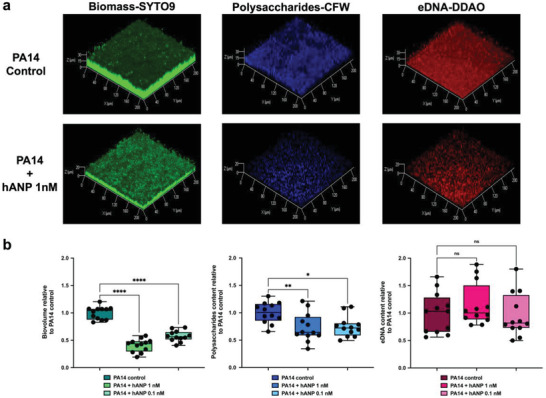
Impact of hANP on *P. aeruginosa* matrix composition. a) 3D shadow representations of the 24 h preformed *P. aeruginosa* PA14 biofilm structures, polysaccharides, and eDNA matrix components, exposed to hANP (1 × 10^−9^ or 0.1 × 10^−9^ m) for 2 h compared to control condition. Bacterial cells within biofilms were stained in green using SYTO9 (left panel). *β*1‐3 and *β*1‐4 polysaccharides were stained in blue using CalcoFluor White (CFW) (central panel). eDNA was stained in red using DDAO (right panel). Image acquisition was performed using CLSM. b) COMSTAT image analyses of bacterial biovolume (left panel), *β*1‐3 and *β*1‐4 polysaccharides (central panel) or eDNA (right panel) matrix components of *P. aeruginosa* PA14 biofilms structures unexposed (control condition) or exposed to hANP (1 × 10^−9^ or 0.1 × 10^−9^ m) for 2 h at 37 °C. Polysaccharides and eDNA values are normalized to biofilm biomass. Data are the result of the analysis of 12 views from four independent biological experiments (*n* = 4). Statistics were achieved by ordinary one‐way ANOVA followed by Dunnett's multiple‐comparison test. Asterisks indicate values that are significantly different as follows: *, *p* < 0.05, **, *p* < 0.01; ****, *p* < 0.0001.

We first speculated that hANP antibiofilm activity is associated with a direct antibacterial activity. However, we observed that exposure of *P. aeruginosa* to hANP at (1 × 10^−6^ or 100 × 10^−9^ m) did not modify the growth kinetic of the bacteria (Figure S3a, Supporting Information), suggesting that hANP has no impact on planktonic cell growth of PA14 strain. These data also fit with the fact that hANP did not affect *P. aeruginosa* membrane fluidity (Figure S3b, Supporting Information). In addition, the proportion of alive bacteria in the biofilms showed no significant difference between hANP‐free and hANP‐treated biofilms (Figure S3c–e, Supporting Information). These data demonstrate that hANP, despite its strong antibiofilm action, is not a bactericidal agent. This led us to hypothesize that hANP alters the composition of the biofilm matrix. In *P. aeruginosa*, extracellular DNA (eDNA) and polysaccharides constitute important components that maintain the cohesion of the biofilm architecture.^[^ ^48^, ^49^ ^]^ To test our hypothesis, polysaccharides and eDNA were evaluated in pre‐established biofilms under hANP exposure using CLSM. Prior to image acquisition, polysaccharides and eDNA matrix components were stained using 7‐hydroxy‐9*H*‐(1,3‐dichloro‐9,9‐dimethylacridin‐2‐one (DDAO) and CalcoFluor White (CFW) fluorescent dyes, and biofilm cells were stained using the SYTO9 green dye. Noticeably, we observed that exposure of preformed biofilm to hANP at 1 × 10^−9^ and 0.1 × 10^−9^ m significantly decreased the relative content of *β*1‐3 and *β*1‐4 polysaccharides (**Figure** **4**a,b). In contrast, the relative abundance of eDNA did not decrease in hANP‐treated biofilms (Figure 4a,b). These results indicate that hANP could trigger a disorganization of the polysaccharide matrix components, which may contribute to its biofilm dispersion activity.

### 2.5 Toward the Elucidation of hANP Mode of Action Involved in Biofilm Dispersion

**Figure 5:**
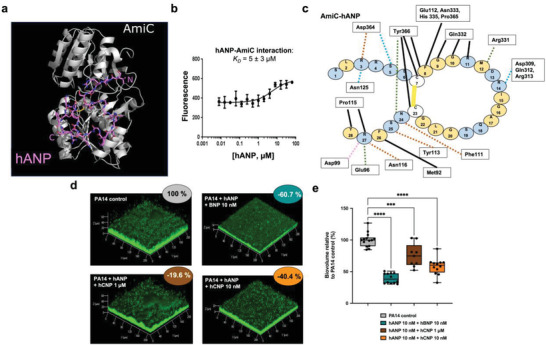
hANP and *P. aeruginosa* AmiC protein binding. a) Docking of hANP to AmiC. Shown is the best pose of hANP docking to the AmiC monomer determined by the FRODOCK server. The peptide binds across the surface where AmiC binds to AmiR. AmiC is shown in the cartoon representation, and hANP is shown in sticks. Colors: AmiC, white; hANP carbon, pink; oxygen, red; nitrogen, blue; sulfur, yellow. Image generated using the PyMOL Molecular Graphics System v2.4.1, Schrödinger, LLC. b) Direct hANP affinity for purified AmiC using MicroScale Thermophoresis. Recombinant AmiC was fluorescently labeled and incubated with varying concentrations of hANP. Data are the mean of three independent experiments. c) Schematic of the AmiC‐hANP interaction. Interactions were determined using LigPlot+ (Figure S4, Supporting Information) and checked manually using PyMOL. The hANP peptide is shown as individual amino acids in circles. AmiC residues interacting with hANP are shown in rectangles. Interactions are colored by type: hydrophobic interactions, black solid line. Disulfide bond, yellow solid line. Hydrogen bond to side chain, orange dashed line. Hydrogen bond to main chain, green dashed line. Salt bridge, pink dashed line. Assigned as hydrophobic by LigPlot but likely hydrogen bonds, blue dashed line. d) 3D shadow representations of the 24 h preformed *P. aeruginosa* PA14 biofilm structures exposed for 2 h at 37 °C to hANP and hBNP (10 × 10^−9^ m each) or to hANP (10 × 10^−9^ m) and hCNP at 1 × 10^−6^ m or 10 × 10^−9^ m compared to control condition (PA14 control; untreated). e) COMSTAT image analyses of biofilms structures of *P. aeruginosa* PA14 control or exposed for 2 h to hANP and hBNP cocktail (10 × 10^−9^ m each) or to hANP (10 × 10^−9^ m) and hCNP at 1 × 10^−6^ or 10 × 10^−9^ m. Data are the result of the analysis of at least nine views from at least three independent biological experiments (*n* = 3). All biofilms were stained with SYTO9 and observed by CLSM. Statistics were achieved by ordinary one‐way ANOVA followed by Dunnett's multiple‐comparison test. Asterisks indicate values that are significantly different as follows: ***, *p* < 0.001; ****, *p* < 0.0001.

We previously showed that the bacterial sensor protein AmiC mediates the action of hCNP on *P. aeruginosa*.^[^ ^44^ ^]^ We tested whether hANP might also act through AmiC first by docking ANP to AmiC (P27017; www.uniprot.org) in silico. hANP showed a clear preference for a loop‐rich region of AmiC (**Figure** **5**a) that is also the binding site for its native partner AmiR. Binding between AmiC and hANP was experimentally confirmed by microscale thermophoresis assays with a *K*_D_ of 5 ± 3 × 10^−6^ m (Figure 5b; see 2.0 ± 0.3 × 10^−6^ m for hCNP^[^ ^44^ ^]^). This is consistent with the van der Waals and hydrogen bonding interactions in the predicted hANP–AmiC complex (Figure 5c). Although CNP binds AmiC,^[^ ^44^ ^]^ it has no effect on biofilm disruption (Figure 2c). Nevertheless, CNP partially blocked the hANP effect when hANP and hCNP were co‐administered on established biofilm (Figure 5d,e). This effect was hCNP concentration‐dependent (Figure 5d,e). As expected, hBNP peptide, which has no affinity for AmiC,^[^ ^44^ ^]^ did not interfere with the hANP antibiofilm activity (Figure 5d,e).

**Figure 6:**
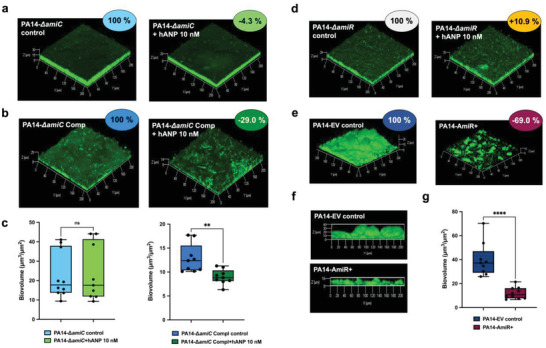
Involvement of *P. aeruginosa ami* operon in hANP antibiofilm effect. a–c) Involvement of the AmiC sensor protein in hANP antibiofilm effect. a) 3D shadow representations of the 24 h old biofilm structures of the mutant strain PA14‐*ΔamiC* control (left panel) or exposed to hANP (2 h; 10 × 10^−9^ m; right panel). b) 3D shadow representations of the 24 h old biofilm structures of the complemented strain PA14‐*ΔamiC* Comp (left panel) or exposed to hANP (2 h; 10 × 10^−9^ m; right panel). c) COMSTAT image analyses of the biofilm structures of *P. aeruginosa* PA14‐*ΔamiC* and PA14‐*ΔamiC* Comp strains, control (blue) or exposed to hANP (green) for 2 h at 37 °C. Data are the result of the analysis of nine views from three independent biological experiments (*n* = 3). d–g) Involvement of the AmiR regulator protein in hANP antibiofilm effect. d) 3D shadow representations of the 24 h old biofilm structures of the mutant PA14‐*ΔamiR* control (left panel) or exposed to hANP (2 h; 10 × 10^−9^ m; right panel). e) 3D shadow representations of the 24 h old biofilm structures of the PA14 strain overexpressing AmiR (AmiR+; right panel) and an empty vector control (PA14 EV control; left panel). f) 3D shadow lateral representations showing the thickness of the 24 h old biofilm structures of the PA14 strain overexpressing (AmiR+; lower panel) and the empty vector control (PA14 EV control; upper panel). g) COMSTAT image analyses of the biofilm structures of both *P. aeruginosa* PA14‐EV and PA14‐AmiR+ strains. Data are the result of the analysis of ten views from three independent biological experiments (*n* = 3). Statistics were achieved by a two‐tailed *t* test. Asterisks indicate values that are significantly different as follows: **, *p* < 0.01; ****, *p* < 0.0001.

To gain further insights into the hANP mode of action on *P. aeruginosa* biofilm dispersion, an *amiC* mutant (PA14‐*ΔamiC*)^[^ ^50^ ^]^ and its complemented strain (PA14‐*ΔamiC* Comp)^[^ ^44^ ^]^ were assessed for biofilm dispersion upon exposure to hANP at 10 × 10^−9^ m for 2 h. hANP‐treatment did not cause any dispersion of the preformed biofilm of the *ΔamiC* mutant (**Figure** **6**a,c). In contrast, the complemented mutant strain recovered, but only partially, its susceptibility to hANP exposure (−29.0 ± 3.7%; *p* < 0.01) (Figure 6b,c), supporting the involvement of AmiC in the hANP‐related biofilm dispersion phenotype. AmiC is known to form a complex with AmiR, an antitermination factor that is released after AmiC activation allowing AmiR to regulate the *ami* operon.^[^ ^51^ ^]^ To ascertain whether the AmiR antitermination regulator also contributes to biofilm dispersion of PA14 upon hANP treatment, pre‐established biofilms of the *amiR*‐deletion mutant (PA14‐*ΔamiR*) were exposed to hANP for 2 h. CLSM images and COMSTAT image analyses clearly showed that *amiR* mutant biofilm was not affected by hANP exposure (10 × 10^−9^ m, 2 h) (Figure 6d). In addition, overproducing AmiR (AmiR+) in the wild‐type strain resulted in decreased biofilm formation after 24 h, observed at both biovolume (−69.0 ± 4.2%; *p* < 0.0001) and thickness levels (−38.7 ± 10.6%) (Figure 6e–g). Interestingly, the biofilm phenotype of PA14‐AmiR+ overproducing strain is similar to that of the wild‐type strain exposed to hANP, indicating that AmiR might possibly be involved in the hANP biofilm dispersion activity. These results further validate the hypothesis that hANP induces PA14 biofilm biomass dispersion in a specific manner. Overall, the data suggest that the AmiC sensor protein represents the bacterial target of hANP, allowing the release of the antitermination regulator AmiR. Together, these findings establish the AmiC‐AmiR complex of *P. aeruginosa ami* pathway as a potential mechanism mediating the hANP biofilm dispersal effect.

## 3 Discussion

Our work presents the first evidence that the human hormone, Atrial Natriuretic Peptide (hANP) has a specific activity against the *P. aeruginosa* biofilm. hANP is competent to both inhibit *P. aeruginosa* biofilm formation, and, more importantly, to strongly disperse established biofilms at extremely low concentrations (1× 10^−9^ to 10 × 10^−9^ m) after 2 h exposure. To our knowledge, no such activity has been reported for a natural product or peptide agent against mature biofilms at the nanomolar level. Previous reports have shown that peptides can disperse biofilms^[^ ^52^, ^53^ ^]^ but these required orders of magnitude of concentration higher than those used in our study and needed at least 24 h exposure. The most effective previous described peptide, the 1018 peptide, disperses *P. aeruginosa* biofilms at 0.5 × 10^−6^ m. This peptide is efficient both against biofilms and planktonic *P. aeruginosa* cells and its own effect seems to be not related to the 1018 peptide sequence since the inverted peptide version possesses the same effect.^[^ ^54^ ^]^

Herein, we also show that hANP did not show an effect on *P. aeruginosa* virulence (Figure S5, Supporting Information), conversely to hCNP, which displays a pro‐virulent activity,^[^ ^43^, ^44^ ^]^ making it a good candidate to tackle biofilms. Many past antibiofilm strategies were focused on the initial steps of biofilm formation, including adhesion and maturation of the biofilm.^[^ ^55^ ^]^ The drug concentrations that are required to inhibit biofilm formation are generally much lower compared to than those used to disrupt established biofilms. As highlighted in the present study, the main interests of hANP are i) its efficacy at low concentration (10 × 10^−9^ m and even 1 × 10^−9^ m), ii) its rapid dispersal effect occurring in short‐duration treatment (2 h, and even 30 min), which is sufficient to disperse around 74% of the biofilm, iii) its synergy with antibiotics of different classes implying its use as a potential adjuvant treatment for *P. aeruginosa*. It should be noted that in case of identified polymicrobial infections (notably *S. aureus* and *P. aeruginosa*), the *S. aureus* locates at the top of the mixed biofilm. It would be recommended to treat the patient with an anti‐*S. aureus* compound before administering ANP alone or with an antibiotic directed against *P. aeruginosa*.

hANP seems to partially breakdown the polysaccharides of the PA14 biofilm matrix (Figure 4) suggesting that hANP might induce the production of polysaccharide‐degrading enzymes. In many Gram‐positive and Gram‐negative bacteria, the biofilm dispersion has been associated with polysaccharide dispersion.^[^ ^23^, ^24^ ^]^ In *P. aeruginosa*, it has been shown that PelA and PslG glycoside hydrolases contribute to biofilm disassembly.^[^ ^48^ ^]^ Since the levels of polysaccharides present in the biofilm matrix differ across *P. aeruginosa* strains, this may explain the distinct dispersal effect of hANP observed on PA14, H103, PAK and the panel of clinical strains. Thus, tackling components of the biofilm matrix might constitute a promising strategy for therapeutics development.

As well as its effect on biofilms, hCNP slightly enhanced bacterial cytotoxicity^[^ ^56^ ^]^ and virulence^[^ ^57^ ^]^ by promoting quorum sensing networks.^[^ ^57^ ^]^ Our data demonstrate that hANP, despite its strong antibiofilm action, was not bactericidal (Figure S3, Supporting Information), did not affect membrane fluidity, and did not enhance virulence (Figure S5, Supporting Information). In addition, compared to the additive effect that hANP has with antibiotics on established biofilms, we observed that hANP did not affect *P. aeruginosa* growth suggesting no impact of hANP on free living bacteria, confirming that hANP only affects the *P. aeruginosa* biofilm lifestyle. Since the current paradigm in the search of antibacterial or antibiofilm agents is to modulate the bacterial virulence and/or biofilm formation rather than killing bacteria (which generally paves the way for the emergence of resistant variants), our data show that hANP meets these criteria. Moreover, the dispersal potential of hANP could greatly reduce the thickness and cohesion of biofilms allowing antibiotics to kill the remaining (or dispersed) planktonic cells. The adjuvant activity that we propose for hANP could apply to different classes of antibiotics independently of their mode of action (e.g., cell wall formation inhibition for imipenem, bacterial membrane disruption for polymyxin, and protein synthesis inhibition for tobramycin).

Previous studies showed that antibiofilm and pro‐virulence activities of hCNP are dependent on the binding of hCNP to AmiC^[^ ^44^ ^]^ in a concentration‐dependent manner.^[^ ^43^ ^]^ In parallel, hCNP and hBNP were characterized for their ability to inhibit biofilm formation through a not yet identified pathway that did not involve AmiC.^[^ ^43^, ^44^ ^]^ Therefore, we evaluated the impact of both hBNP and hCNP on established biofilms. Unlike hANP, these two peptides were unable to disrupt established biofilms, an expected result for hBNP since it has no affinity for AmiC.^[^ ^44^ ^]^ It is surprising that hCNP shows no effect since it binds AmiC with a micromolar affinity.^[^ ^44^ ^]^ In silico superposition of the 3D structures of AmiC and known NP receptors defined AmiC as an equivalent of the human NP receptor subtype C (hNPR‐C).^[^ ^44^ ^]^ hANP binds to AmiC with a *K*_D_ around 5 × 10^−6^ m in vitro (Figure 5b), in the same order as observed for hCNP.^[^ ^44^ ^]^ Interestingly, pharmacological studies have shown that hNPR‐C preferentially binds hANP compared to hCNP while displaying a low affinity for hBNP.^[^ ^58^ ^]^ Altogether, these data suggest that AmiC could display a strong affinity for hANP compared to hCNP in vivo in the bacterial cell in contrast to our in vitro observations obtained by microscale thermophoresis. In addition, comparing the putative binding areas of hCNP^[^ ^44^ ^]^ and hANP (this study) to AmiC, it is possible that the AmiC amino acids involved in these interactions are not identical. This suggests a slight shift of the binding that could explain a difference in term of AmiR release, after NP binding on AmiC. The binding of AmiC to AmiR occurs across a loop‐rich surface of AmiC, with specific interactions formed by the Lys89, Glu96*, Phe111*, Tyr113*, Ile151, Glu155, His158, Gln332*, Asp364*, and Tyr366*.^[^ ^59^ ^]^ Our in silico molecular docking suggests that hANP could bind specifically to AmiC (Figure 5c). Six of the key AmiR‐binding residues are predicted to interact with hANP (indicated by asterisks above). Moreover, AmiC Phe111, which has an important role in AmiR sequestration by AmiC,^[^ ^59^ ^]^ is predicted to interact with Asn24 of hANP, whereas hCNP is predicted to bind Phe111 through the Asp12.^[^ ^44^ ^]^ These data suggest different interactions between AmiC and hANP or hCNP that could potentially trigger a different mechanism of AmiR release. Exposure of *P. aeruginosa* biofilms to hANP and hCNP simultaneously prevented the hANP dispersal effect (Figure 5d). This suggests that hCNP can act as an hANP antagonist or competitor for its binding to AmiC. This observation is reinforced by the dose‐dependence of hCNP's antagonism of the hANP effect (Figure 5d). By contrast, hBNP, which displays no affinity to AmiC, did not influence the hANP dispersal effect (Figure 5d). Finally, the precise site of interaction between AmiC and hANP should be further investigated even if there is evidence that PA14_64270, a protein presenting 29% of similarity with AmiC, could be involved in this interaction as it was demonstrated for hCNP and AmiC association.^[^ ^37^, ^44^ ^]^

Analysis of the in silico putative hANP binding to AmiC suggests that hANP could compete with AmiR, favoring the de‐sequestration of AmiC by AmiR. This suggested that AmiR might also be involved in the hANP effect on biofilm dispersion. We observed that *amiR* deletion mutant biofilm was not susceptible to dispersion by hANP, suggesting that the AmiC/AmiR complex might potentially mediate the antibiofilm activity of hANP. Overproduction of AmiR strongly impaired biofilm formation, thus mimicking the antibiofilm effect of hANP. It has previously been observed that a *P. aeruginosa* strain overexpressing the AmiE amidase (the final product of the *ami* operon) presented an altered biofilm after 24 h of growth,^[^ ^60^ ^]^ also mimicking the effect of hANP on established biofilms (our study). These findings support the hypothesis that the *ami* operon products contribute to biofilm regulation in *P. aeruginosa*. This hypothesis is strongly supported by studies showing that mRNA expression levels of the whole *ami* operon genes are highly overexpressed in biofilms compared to bacteria in the planktonic state or in dispersed cells.^[^ ^61^ ^]^ In addition, the abundance of AmiE protein appears to be altered during biofilm formation, being particularly high in 96 h old biofilms, consistent with a role in dispersion.^[^ ^62^ ^]^ In contrast, AmiC abundance shows low levels of modulation during biofilm formation compared to AmiE.^[^ ^62^ ^]^ Interestingly, during planktonic growth no abundance variations in AmiE or in other *ami*‐encoded proteins were observed.^[^ ^62^ ^]^ A second study by the same group confirmed that AmiE abundance is strongly enhanced (8.4‐fold) when bacteria are grown in biofilm (96 h), whereas AmiR is over‐accumulated only during the first 24 h of biofilm formation.^[^ ^63^ ^]^ Altogether, these data suggest that in addition to its role in nitrogen/carbon metabolism, the function of the products of the *ami* operon in *P. aeruginosa* biofilm regulation is probably under‐evaluated. Our investigation into the mechanism of hANP action on established biofilms further supports this supposition. Further research is being undertaken to investigate the key role of the AmiR antitermination regulator and all members of the *ami* operon in both the regulation of biofilm formation and the maturation/dispersion of *P. aeruginosa* biofilms. It will be interesting to elucidate the potential role of AmiR in the direct or indirect regulation of c‐di‐GMP signaling pathway during *P. aeruginosa* biofilm maturation and dispersion, through NO donor regulation,^[^ ^64^ ^]^ motility modification, and siderophore synthesis.

## 4 Conclusion

In summary, we propose hANP as a potent dispersal agent against *P. aeruginosa* biofilms even at very low concentrations. The hANP human hormone has a higher potential compared to other NPs either on its own, or as an antibiotic adjuvant to eradicate *P. aeruginosa* biofilms. Our study also suggests that members of the *ami* operon mediate the hANP antibiofilm effect, revealing their contribution to biofilm dispersion in *P. aeruginosa*. In addition, it is important to note that the antibiofilm activity of hANP is not associated with bacterial virulence enhancement. This suggests that the risk of *P. aeruginosa* responding to hANP treatment by developing resistance is low. Moreover, the high potential of hANP as a therapeutic agent to disperse *P. aeruginosa* biofilms is reinforced by the fact that hANP is not cytotoxic toward lung cells in vitro and did not modify hERG cardiac channel conductance, the first step to validate the absence of cardiac toxicity of a future drug (Figure S6, Supporting Information). hANP also exercises i) a positive effect during acute lung injury induced by LPS,^[^ ^65^, ^66^ ^]^ ii) antifibrotic activity,^[^ ^67^ ^]^ and iii) anti‐inflammatory effect, notably in the lung.^[^ ^68^, ^69^ ^]^ For all these reasons, we propose hANP as a promising powerful antibiofilm weapon against established *P. aeruginosa* biofilms in chronic infections.

## 5 Experimental Section

### Bacterial Strains, Media, and Growth Conditions

The bacterial strains used in this study are listed in Table S1 in the Supporting Information. The *Pseudomonas aeruginosa* PA14 wild‐type strain was from Harvard Medical School (Boston, MA)^[^ ^50^ ^]^ and kindly provided by the Biomerit Research Center (Univ. Cork, Ireland). *P. aeruginosa* H103 is a prototroph of PAO1 wild‐type strain.^[^ ^70^ ^]^ *P. aeruginosa* PAK is a nonmucoid clinical strain.^[^ ^71^ ^]^ The MUC‐N1, MUC‐N2, MUC‐P4, and MUC‐P5 *P. aeruginosa* strains were isolated from sputum samples of adult cystic fibrosis patients followed at the Centre de Référence Contre la Mucoviscidose (CRCM), Centre Hospitalier Universitaire (CHU) of Nantes (France).^[^ ^72^ ^]^ The *P. aeruginosa* clinical strains CF 8.19, CF 9.19 were isolated from sputum of cystic fibrosis patients (CF 8.19 and CF 9.19) (CNR résistance aux antibiotiques CHU Besançon, France). PAL 0.1 and PAL 1.1 were isolated from the airways of an intensive care unit (ICU) patient (Lille CHU hospital, France), with ventilator‐associated pneumonia (PAL1.1), or isolated from blood cultures of an ICU patient with sepsis PAL0.1.^[^ ^73^ ^]^ All bacterial strains were grown at 37 °C in Luria Bertani (LB) medium. To this end, overnight bacterial cultures grown aerobically at 37 °C in LB broth in a rotary shaker at 180 rpm were diluted to an OD_580nm_ value of 0.08. When required (for plasmid maintenance), *Escherichia coli* strains were grown in the presence of ampicillin (Ap; 100 µg mL^−1^). *P. aeruginosa* strains were grown in LB liquid cultures in the presence of carbenicillin (Cb; 300 µg mL^−1^) on LB agar (1.5%) plates containing Cb (600 µg mL^−1^). The antibiotics stock solutions used in this study were sterilized by filtration through 0.22 µm filters, aliquoted into daily‐use volumes and kept at −20 °C. Each set of experiments was performed at least three times.

### Construction of *P. aeruginosa* PA14‐*ΔamiR* Deletion Mutant and PA14‐AmiR+ Strain Overexpressing the AmiR Protein

A strain unable to express the AmiR protein was constructed using the procedure described by Quénée et al.^[^ ^74^ ^]^ The upstream and downstream *amiR* flanking regions were PCR (polymerase chain reaction) amplified using the primers listed in Table S2 in the Supporting Information. The two fragments were ligated by PCR and the resulting amplicon was digested with *Sac*I and *Hind*III and cloned in the pEX100T‐link suicide vector. The plasmid was introduced into the *E. coli* S17.1 helper strain and transferred by conjugation into *P. aeruginosa* PA14. Carbenicillin‐resistant PA14 colonies grown on Pseudomonas Isolation Agar (PIA) were counter‐selected on LB agar plate supplemented with 5% w/v sucrose. Double recombinants were selected for their carbenicillin sensitivity and their sucrose resistance. The resulting deletion and absence of ORF frameshift was checked by sequencing (Sanger sequencing services, Genewiz).

The *P. aeruginosa* PA14 strain overexpressing the AmiR protein (AmiR+) was obtained by transformation with the pBBR1‐MCS4 plasmid^[^ ^75^ ^]^ containing the *amiR* gene. The ORF of *amiR* was amplified from *P. aeruginosa* PA14 chromosomal DNA by using the primer pairs amiR‐*Xho* I‐F and amiR‐*Xba* I‐R which contain *Xho*I end *Xba*I restriction sites at their respective 5’ ends (Table S2, Supporting Information). PCR entailed 30 cycles of 30 s at 95 °C, 30 s at 60 °C, and 1 min at 72 °C. The PCR fragment was purified, digested with *Xho*I and *Xba*I, and cloned into the broad‐host‐range expression vector pBBR1‐MCS4, to create pBBR1‐MCS4‐AmiR. Finally, the plasmids pBBR1‐MCS4 (empty vector) and pBBR1‐MCS4‐AmiR were transferred by electroporation separately into the *P. aeruginosa* PA14 strain. The resulting PA14‐EV and PA14‐AmiR+ strains (Table S1, Supporting Information) were then confirmed by PCR and DNA sequencing (Sanger sequencing services, Genewiz).

### Test Substances

The human Atrial Natriuretic Peptide (hANP) was purchased from Calbiochem Merck (United States) and both human BNP (Brain Natriuretic Peptide) and human CNP (C‐type Natriuretic Peptide) were purchased from Alfa Aesar (Switzerland). Stock solutions at 1 mg mL^−1^ were prepared in ultrapure water of each peptide and stored at −20 °C until use.

### Flow Cell Biofilm Assays under Hydrodynamic Conditions

For biofilm formation assays, overnight cultures of *P. aeruginosa* strains grown in LB medium at 37 °C for 18 h with shaking (180 rpm) were sub‐cultured using an inoculum at an OD_580_ value of 0.08 in the same medium and grown for 2 h at 37 °C with shaking (180 rpm). When required, hANP was added at 500 × 10^−9^, 100 × 10^−9^, or 10 × 10^−9^ m final concentration and bacteria were grown for additional 3 h at 37 °C under agitation (180 rpm). Bacterial cells were then washed and adjusted to OD_580_ = 0.1 in 0.9% w/v NaCl. The bacterial suspensions were then used to study biofilm formation under hydrodynamic conditions at 37 °C in a three‐channel flow cell as described by Bazire et al.^[^ ^76^ ^]^ Briefly, each channel of the flow cell was inoculated with 300 µL of bacterial suspension. A 2 h attachment step was performed without flow. After 2 h, each channel was then pumped with a 2.5 mL h^–1^ flow of LB medium, without or with hANP at the required concentration during 24 h. Next, biofilm cells were stained and then observed by CLSM.

For biofilm dispersion experiments, overnight cultures of *P. aeruginosa* strains were performed in LB medium at 37 °C for 18 h with shaking (180 rpm). Bacterial cells were then washed and adjusted to OD_580_ = 0.1 in 0.9% w/v NaCl. The bacterial suspensions were then used to form a biofilm for 24 h under hydrodynamic conditions at 37 °C. Briefly, each channel of the flow cell was inoculated with 300 µL of bacterial suspension. A 2 h attachment step was performed without flow. After 2 h, each channel was then pumped with a 2.5 mL h^–1^ flow rate of LB medium during 24 h. Next, the 24 h old biofilm of *P. aeruginosa* was exposed for 2 h (or 30 min) to 300 µL of studied compounds (i.e., peptides, antibiotics, or a combination of peptide + antibiotics) or 300 µL of ultra‐pure distilled water (control condition), added to each channel of the flow cell and without flow. Prior to image acquisition, biofilm cultures were then rinsed with LB medium using 2.5 mL h^–1^ flow rate for 15 min. Finally, biofilm cells were stained and then observed by CLSM.

### Confocal Laser Scanning Microscopy

The CLSM observations of biofilms were performed using a Zeiss LSM710 microscope (Carl Zeiss Microscopy, Oberkochen, Germany) using a x40 oil immersion objective. Bacteria into the biofilm were stained with 5 × 10^−6^ m of SYTO9 green‐fluorescent dye (Invitrogen, Carlsbad, CA). Biofilm matrix components were stained using fluorescent dyes. To assess the eDNA relative abundance, 1 × 10^−6^ m of DDAO (Invitrogen, Carlsbad, CA) was used. The polysaccharides *β*1‐3 and *β*1‐4 were labeled with CalcoFluor White M2R at 200 µg mL^−1^ (Sigma‐Aldrich, USA). eDNA and polysaccharides values were normalized to biofilms’ biomass. The Live/Dead BacLight kit (Invitrogen, Carlsbad, CA) was used to examine bacterial viability in biofilms according to manufacturer instructions. Images were taken every micrometer throughout the whole biofilm depth. For visualization and processing of 3D image, the Zen 2.1 SP1 software (Carl Zeiss Microscopy, Oberkochen, Germany) was used. Quantitative analyses of images stacks were performed using the COMSTAT software (http://www.imageanalysis.dk/).^[^ ^77^ ^]^ At least three image stacks from at least three independent experiments were used for each analysis.

### Molecular Docking

The hANP peptide from the ANP‐ANP receptor structure (PDB ID: 7BRH, chain C; http://doi.org/10.2210/pdb7BRH/pdb) was extended to include all N‐terminal residues using COOT v.0.9.4.^[^ ^78^ ^]^ hANP was docked to either the AmiC‐acetamide dimer (PDB ID: 1PEA, with the dimer formed around a crystallographic axis^[^ ^79^ ^]^); or an AmiC monomer from the complex with AmiR (PDB ID: 1QO0, chain A^[^ ^59^ ^]^); using the FRODOCK 2.0 server^[^ ^80^ ^]^ (http://frodock.chaconlab.org/), using default parameters. The top scoring docking poses were energy minimized with YASARA v.20.12.24^[^ ^81^ ^]^ using the em_runclean macro with the AMBER15FB force field.^[^ ^82^ ^]^

### Microscale Thermophoresis (MST)

The MST experiments were carried out as previously described.^[^ ^44^ ^]^ Briefly, AmiC protein was labeled using the RED‐NHS labeling kit (NanoTemper Technologies). The labeling reaction was performed according to the manufacturer's instructions in the supplied labeling buffer using a 20 × 10^−6^ m protein concentration and a molar dye:protein ratio ≈2:1, at RT for 30 min. Unreacted dye was removed with the supplied unreact removal columns equilibrated with MST buffer (50 × 10^−3^ m Tris‐HCl pH 7.5, 150 × 10^−3^ m NaCl, 10 × 10^−3^ m MgCl_2_). The label:protein ratio was determined using photometry at 650 and 280 nm. Typically, a ratio of 0.8 was achieved.

hANP was dissolved in MST buffer supplemented with 0.05% Tween‐20. A series of 1:1 dilutions were prepared in the identical buffer, producing ligand concentrations ranging from 13 × 10^−9^ to 434 × 10^−6^ m (hANP). For thermophoresis, each ligand dilution was mixed with one volume of AmiC, which led to a final concentration of AmiC of 50 × 10^−9^ m and final ligand concentrations at half of the ranges above. Instrument parameters were adjusted with 50% light‐emitting diode power and 20% MST power. Data of three independently pipetted measurements were analyzed (NT.Analysis software version 1.5.41, NanoTemper Technologies) using the signal from Thermophoresis + T‐Jump.

### Statistical Analyses

Statistical significance was evaluated using Prism GraphPad software version 9.0. The data were statistically analyzed using ordinary one‐way analysis of variance (ANOVA) followed by Dunnett's multiple comparison test, or unpaired (two sample) two‐tailed *t* test to calculate *p* values. Experiments were performed with at least three biological independent replicates and results were displayed as means ± SEMs (standard error of the means).

## Conflict of Interest

The authors declare no conflict of interest.

## Supporting information

Supporting Information

## Data Availability

The data that support the findings of this study are available from the corresponding author upon reasonable request.
