# Supplementary material for: Pseudomonas aeruginosa Biofilm Dispersion by the Human Atrial Natriuretic Peptide
Source: Adv Sci (Weinh). 2022 Jan 14;9(7):2103262. doi: 10.1002/advs.202103262 (PMC8895129; doi:10.1002/advs.202103262)
Supplement: Supplementary file 1 — Supporting Information [file ADVS-9-2103262-s001.pdf]

ADVANCED SCIENCE
Open Access

Supporting Information

for *Adv. Sci.*, DOI: 10.1002/advs.202103262

Pseudomonas aeruginosa Biofilm Dispersion by the Human Atrial Natriuretic Peptide

Mélissande Louis[1], Thomas Clamens[1], Ali Tahrioui[1], Florie Desriac[1,2], Sophie Rodrigues[1], Thibaut Rosay[1], Nicholas Harmer[3], Suraya Diaz[3], Magalie Barreau[1], Pierre-Jean Racine[1], Eric Kipnis[4], Teddy Grandjean[4], Julien Vieillard[5], Emeline Bouffartigues[1], Pierre Cornelis[1], Sylvie Chevalier[1], Marc G.J. Feuilloley[1] and Olivier Lesouhaitier[1]*

Supplementary Information

Pseudomonas aeruginosa Biofilm Dispersion by the Human Atrial Natriuretic Peptide

Mélissande Louis[1], Thomas Clamens[1], Ali Tahrioui[1], Florie Desriac[1,2], Sophie Rodrigues[1], Thibaut Rosay[1], Nicholas Harmer[3], Suraya Diaz[3], Magalie Barreau[1], Pierre-Jean Racine[1], Eric Kipnis[4], Teddy Grandjean[4], Julien Vieillard[5], Emeline Bouffartigues[1], Pierre Cornelis[1], Sylvie Chevalier[1], Marc G.J. Feuilloley[1] and Olivier Lesouhaitier[1]*

[1]*Laboratory of Microbiology Signals and Microenvironment LMSM EA 4312, University of Rouen Normandy, 27000 Evreux, France*

[2]*Normandie Univ, UNICAEN, Unité De Recherche Risques Microbiens U2RM, 14000 Caen, France*

[3]*School of Biosciences, University of Exeter, Exeter, EX4 4QD, UK*

[4]*Univ. Lille, CNRS, Inserm, CHU Lille, Institut Pasteur de Lille, U1019-UMR9017-CIIL-Centre d'Infection et d'Immunité de Lille, Lille, France, University Lille, F-59000, Lille, France.*

[5]*Normandie Univ, UNIROUEN, INSA Rouen, CNRS, COBRA (UMR 6014), 27000 Evreux, France*

* Corresponding author: Olivier Lesouhaitier (olivier.lesouhaitier@univ-rouen.fr)

Supplementary Figures 1-8
Supplementary Tables 1-2
Supplementary Methods
Supplementary References

Supplementary Figures 1-8

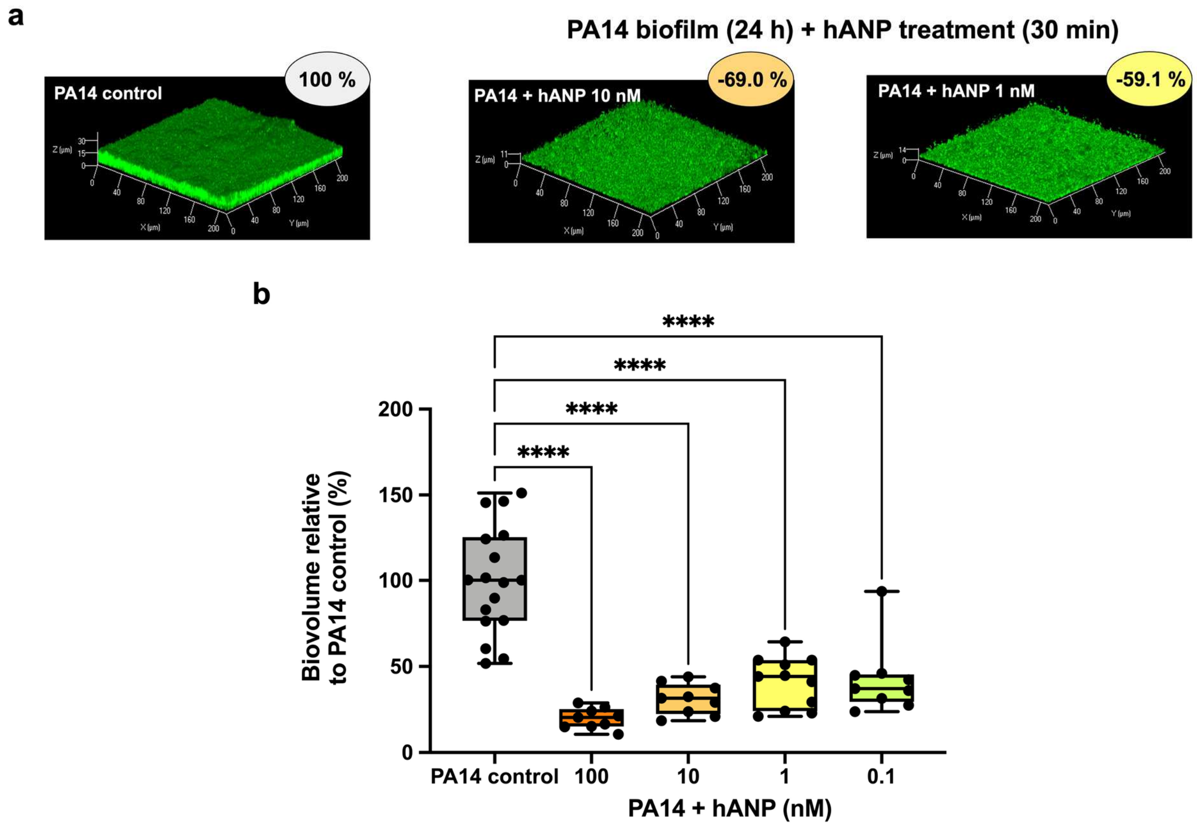

Fig. 1. Effect of short exposure (30 min) to Atrial Natriuretic Peptide (hANP) on *P. aeruginosa* PA14 pre-formed biofilms in flow cell system. a, 3D-shadow representations of 24 h-old pre-formed *P. aeruginosa* PA14 biofilm structures unexposed (control condition) or exposed (30 min) to hANP (10 nM or 1 nM). **b,** COMSTAT image analyses of biofilms structures of *P. aeruginosa* PA14 control or exposed for 30 min to hANP (100 nM, 10 nM, 1 nM or 0.1 nM). Data are the result of the analysis of 9 views from three independent biological experiments (hANP 100 nM), 9 views from three independent biological experiments (hANP 10 nM), 28 measurements from nine independent experiments (hANP 1 nM), and 9 views from three independent biological experiments (hANP 0.1 nM). Statistics were achieved by ordinary one-way ANOVA followed by Dunnett's multiple-comparison test. Asterisks indicate values that are significantly different as follows: ****, $P < 0.0001$.

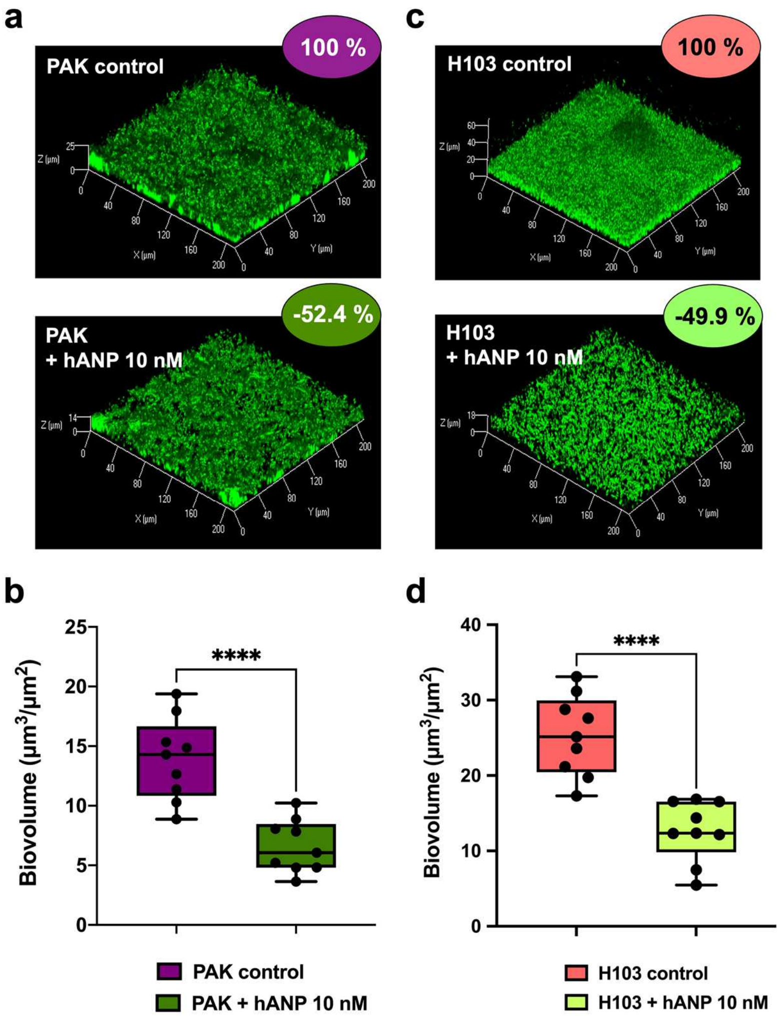

Fig. 2. Impact of Atrial Natriuretic Peptide (hANP) on pre-formed biofilm of *P. aeruginosa* PAK and H103 strains. a, 3D-shadow representations of the 24 h pre-formed *P. aeruginosa* PAK biofilm untreated (control condition) or treated to hANP at 10 nM for 2 h. **b,** COMSTAT image analyses of biofilms of *P. aeruginosa* PAK upon exposure to hANP (10 nM). **c,** 3D-shadow representations of the 24 h pre-formed *P. aeruginosa* H103 biofilm untreated (control condition) or treated to hANP at 10 nM for 2 h. **d,** COMSTAT images analyses of biofilm structures of *P. aeruginosa* H103 upon exposure to hANP (10 nM). Data are the result of the analysis of three independent biological assays (n=3) with three views each. Statistics were achieved by a two-tailed *t* test. Asterisks indicate values that are significantly different as follows: ****, $P < 0.0001$.

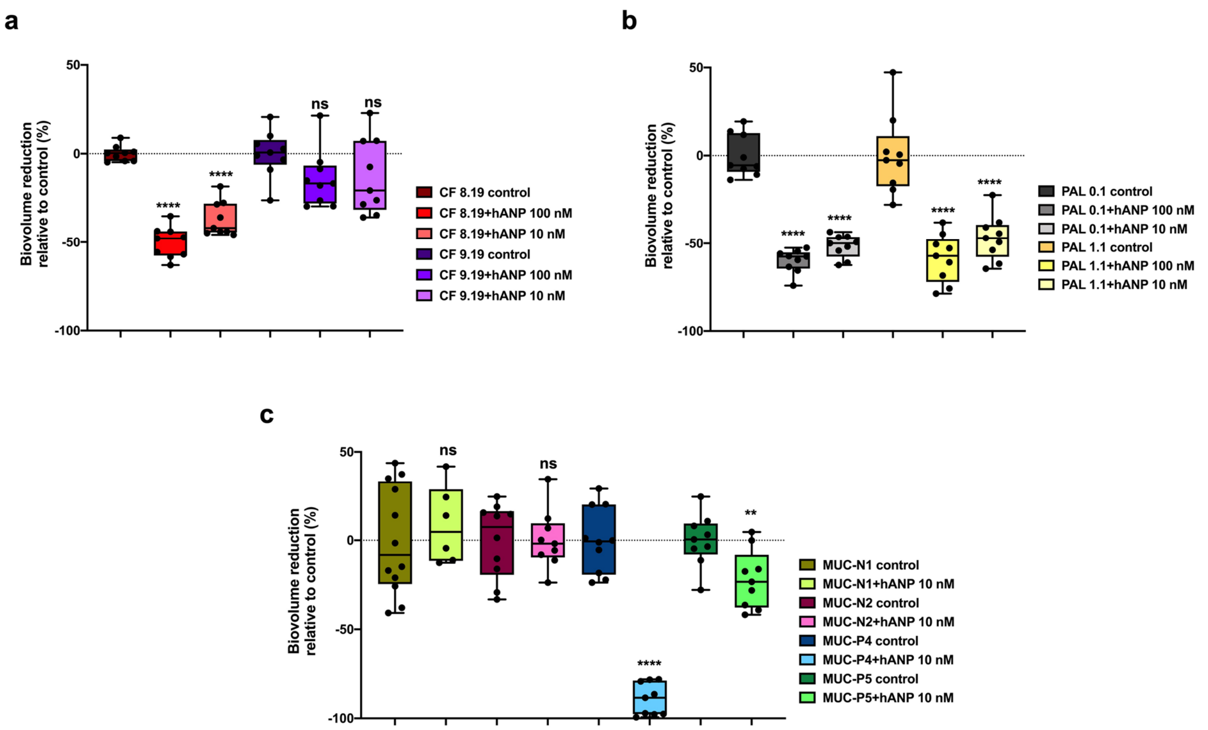

Fig. 3. Effect of Atrial Natriuretic Peptide (hANP) on pre-formed biofilm of *P. aeruginosa* clinical strains. a, COMSTAT images analyses of 24 h established biofilm structures of *P. aeruginosa* CF 8.19 and CF 9.19 clinical strains upon exposure (2h) to hANP at 100 nM and 10 nM. Data are the result of the analysis of three independent biological assays (n=3) with three views each. Statistics were achieved by ordinary one-way ANOVA followed by Dunnett's multiple-comparison test. Asterisks indicate values that are significantly different as follows: ****, $P < 0.0001$; ^{ns}, not significant ($P \geq 0.05$). **b,** COMSTAT image analyses of 24 h established biofilms of *P. aeruginosa* PAL 0.1 and PAL 1.1 clinical isolates upon exposure (2h) to hANP at 100 nM and 10 nM. Data are the result of the analysis of three independent biological assays (n=3) with three views each. Statistics were achieved by ordinary one-way ANOVA followed by Dunnett's multiple-comparison test. Asterisks indicate values that are significantly different as follows: ****, $P < 0.0001$. **c,** COMSTAT image analyses of 24 h pre-formed MUC-N1, MUC-N2, MUC-P4, and MUC-P5 *P. aeruginosa* clinical strains untreated (control condition) or treated to hANP at 10 nM for 2 h. Data are the result of the analysis of three independent biological assays (n=3) with at least three views each unless for MUC-N1 isolate for which only two independent biological assays were performed. Statistics were achieved by a two-tailed *t* test. Asterisks indicate values that are significantly different as follows: **, $P < 0.01$; ****, $P < 0.0001$; ^{ns}, not significant ($P \geq 0.05$).

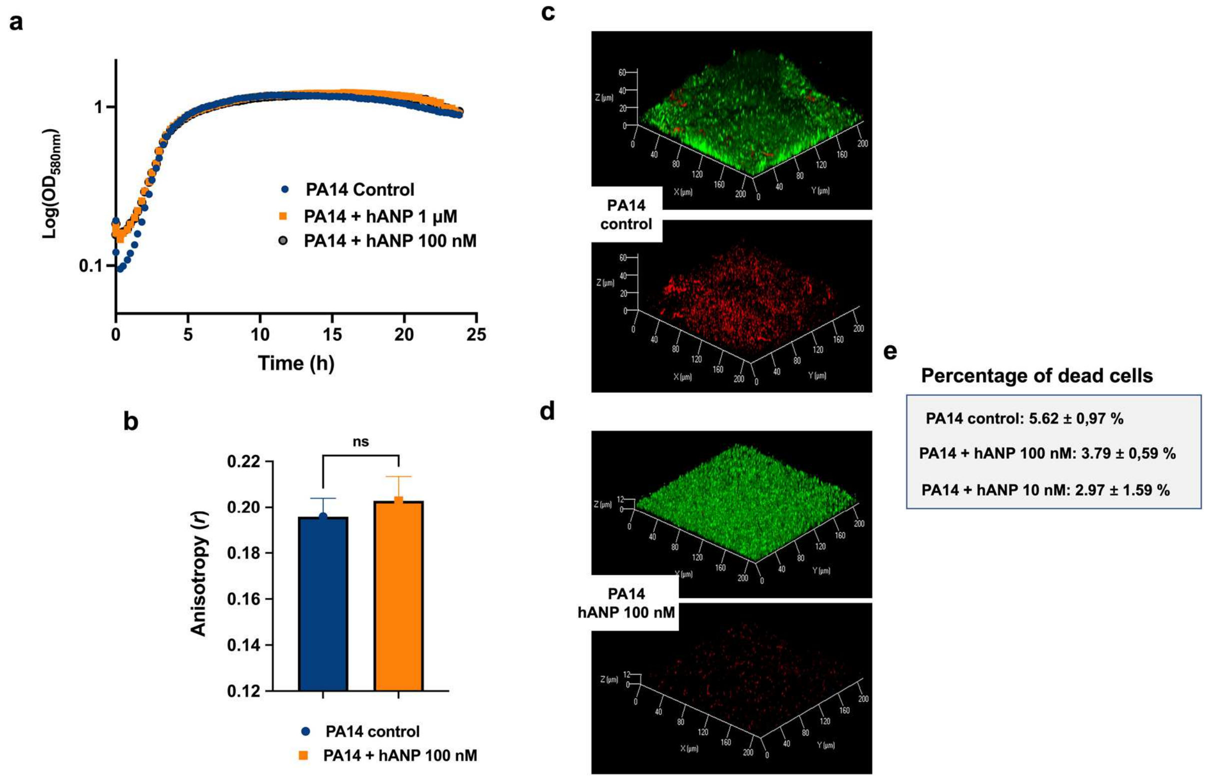

Fig. 4. Effect of Atrial Natriuretic Peptide (hANP) on *P. aeruginosa* cell growth and viability, and membrane fluidity. a, Growth of PA14 under hANP exposure. Culture growth curves were measured at OD_{580nm}. Absorbance was recorded every 15 min for 24 h. Curves are color-coded as follow: PA14 strain used as control (blue), PA14 exposed to hANP (1 µM) (orange) and PA14 exposed to hANP (0.1 µM) (grey). Results are the mean of eight replicates from three independent experiments. **b,** Membrane fluidity of *P. aeruginosa* PA14 exposed to hANP. The fluorescence anisotropy values (*r*) for PA14 strain used as control (blue bar) and PA14 exposed to hANP at 0.1 µM (orange bar). The error bars represent the standard error of the means (SEMs) and are the result of the analysis of three independent biological assays with eight measurements each. Statistics were achieved by a two-tailed *t* test. ^{ns}, not significant ($P \geq 0.05$). **c, d,** and **e,** Effect of hANP on 24 h-old biofilm cell viability. Quantification of alive and dead cells was assessed using the Live/Dead BacLight kit.

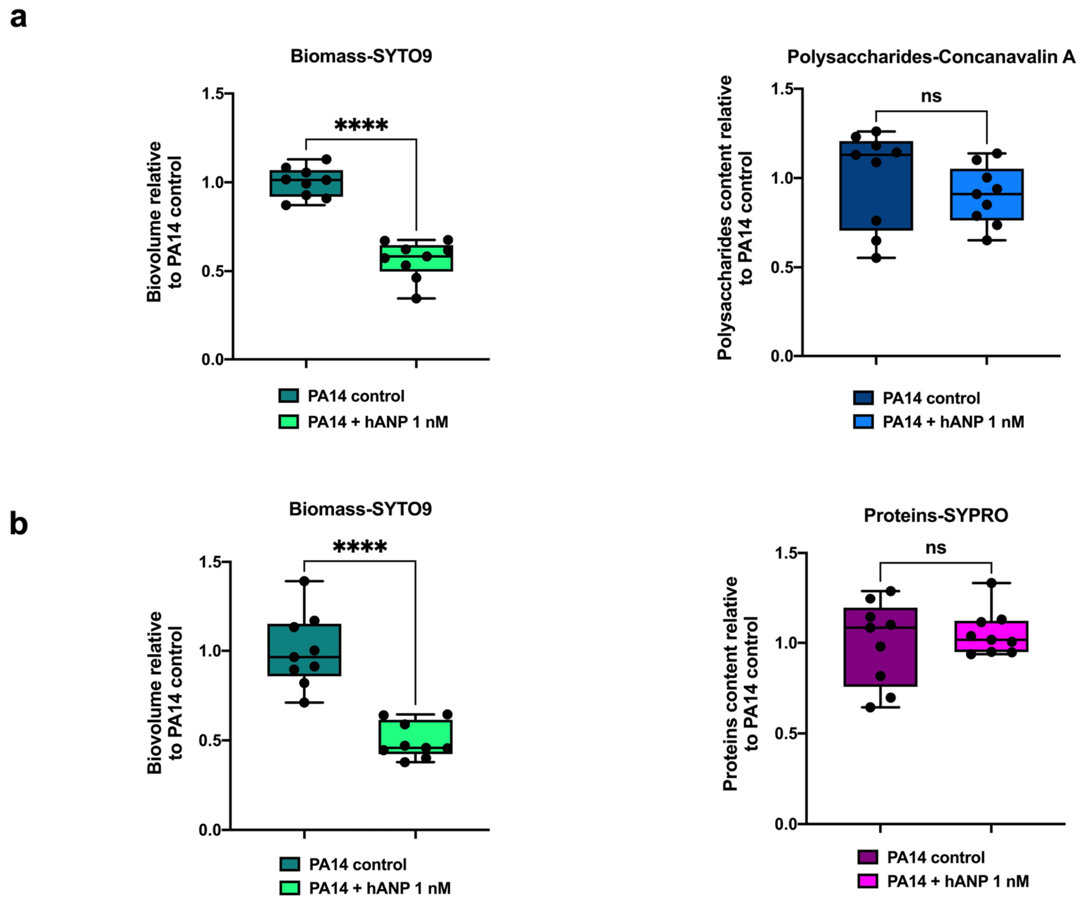

Figure 4. Impact of ANP on *P. aeruginosa* matrix composition. a, COMSTAT image analyses of bacterial biovolume (left panel) and α polysaccharides (right panel) matrix components of *P. aeruginosa* PA14 biofilms structures unexposed (control condition) or exposed to ANP (1 nM) for 2 h at 37°C. **b,** COMSTAT image analyses of bacterial biovolume (left panel) and proteins (right panel) matrix components of *P. aeruginosa* PA14 biofilms structures unexposed (control condition) or exposed to ANP (1 nM) for 2 h at 37°C. Bacterial cells within biofilms were stained using SYTO9. α polysaccharides were stained using Concanavalin A. Proteins were stained using SYPRO ruby. Polysaccharides and Proteins values are normalized to biofilm biomass. Data are the result of the analysis of nine views from three independent biological experiments (n=3). Statistics were achieved by a two-tailed *t* test. Asterisks indicate values that are significantly different as follows: ****, $P < 0.0001$; ^{ns}, not significant ($P \geq 0.05$).

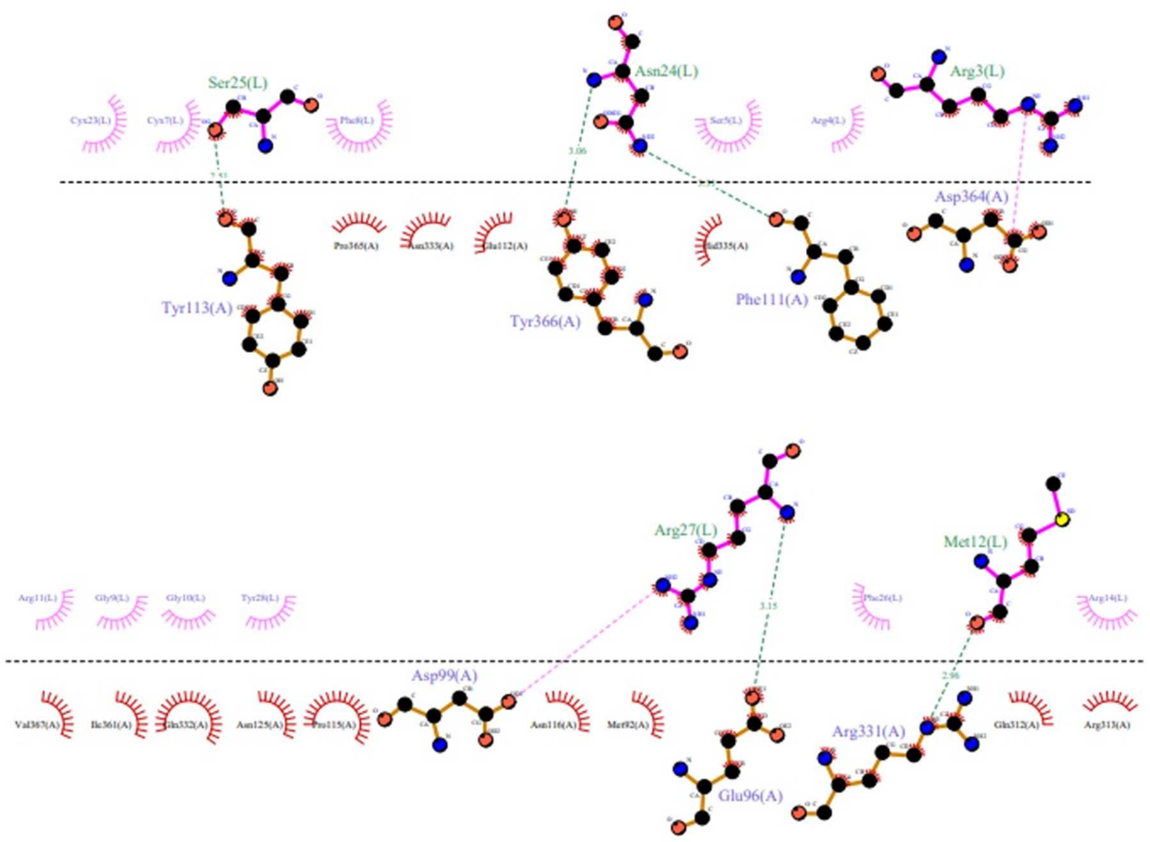

Fig. 6. Ligplot image representing the interaction between hANP and AmiC. Interactions between the human ANP peptide and the AmiC *P. aeruginosa* protein were determined using LigPlot+ (Laskowski and Swindells, 2011). **Key:** green dotted lines: hydrogen bonds between a side chain and main chain; pink dotted lines: salt bridge; pink dashed arc: hydrophobic interactions (hANP); red dashed arc: hydrophobic interactions (AmiC). The direction of the arcs indicates the residues on the other partner that the hydrophobic interactions are formed between.

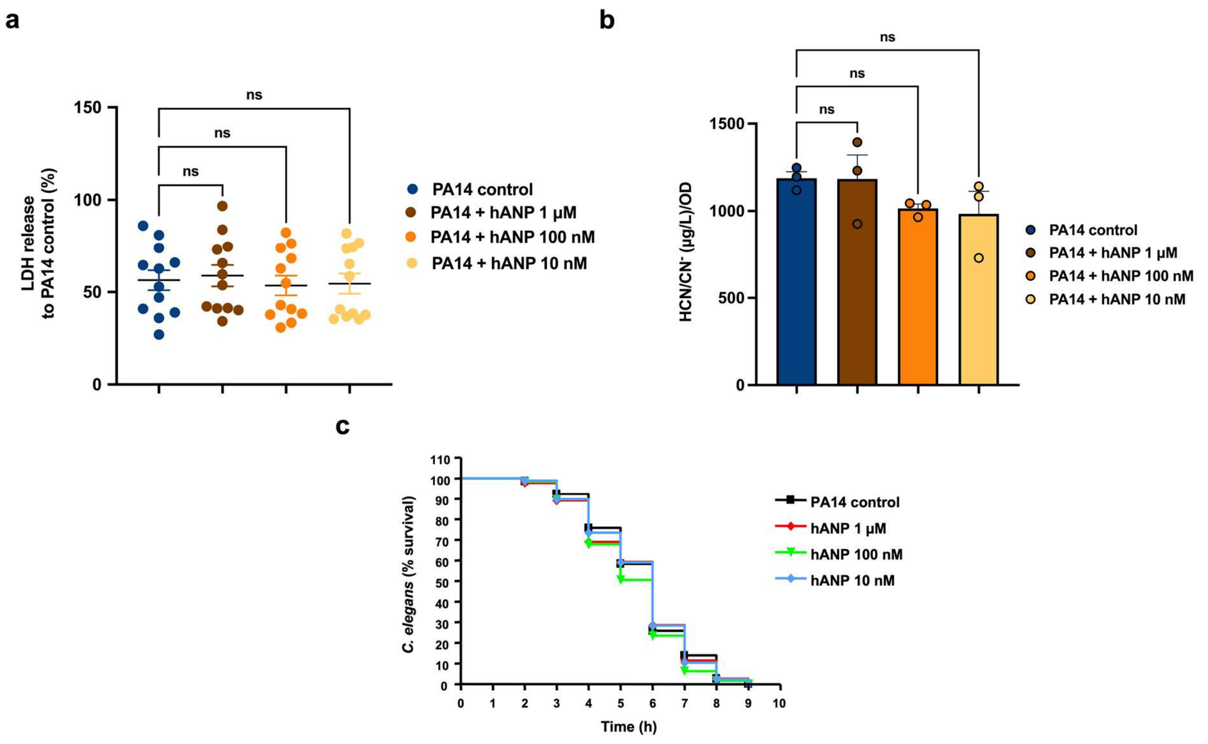

Fig. 7. Impact of Atrial Natriuretic Peptide (hANP) on *P. aeruginosa* virulence. a, Percentage of lactate dehydrogenase (LDH) released by A549 lung cells after 5 h infection with *P. aeruginosa* PA14 unexposed (control; blue bar) or exposed to various concentrations of hANP (orange bars). Data are the result of the analysis of three independent biological assays with four replicates each. Statistics were achieved by ordinary one-way ANOVA followed by Dunnett's multiple-comparison test. ^{ns}, not significant ($P \geq 0.05$). **b,** HCN/CN^{-} production in PA14 control condition (dark blue bar), PA14 exposed to hANP at 1 µM (brown bar), 100 nM (orange bar), and 10 nM (light orange bar). Data are the result of the analysis of three independent biological assays with three replicates each. Statistics were achieved by ordinary one-way ANOVA followed by Dunnett's multiple-comparison test. ^{ns}, not significant ($P \geq 0.05$). **c,** Kaplan-Meier survival plots of *C. elegans* challenged by PA14 unexposed to hANP (black squares; n = 194) or exposed to hANP at 1 µM (red diamonds; n = 228), 0.1 µM (inversed green triangle; n = 192) or 10 nM (blue diamonds; n = 173). For each condition, the median survival was obtained at 6 h.

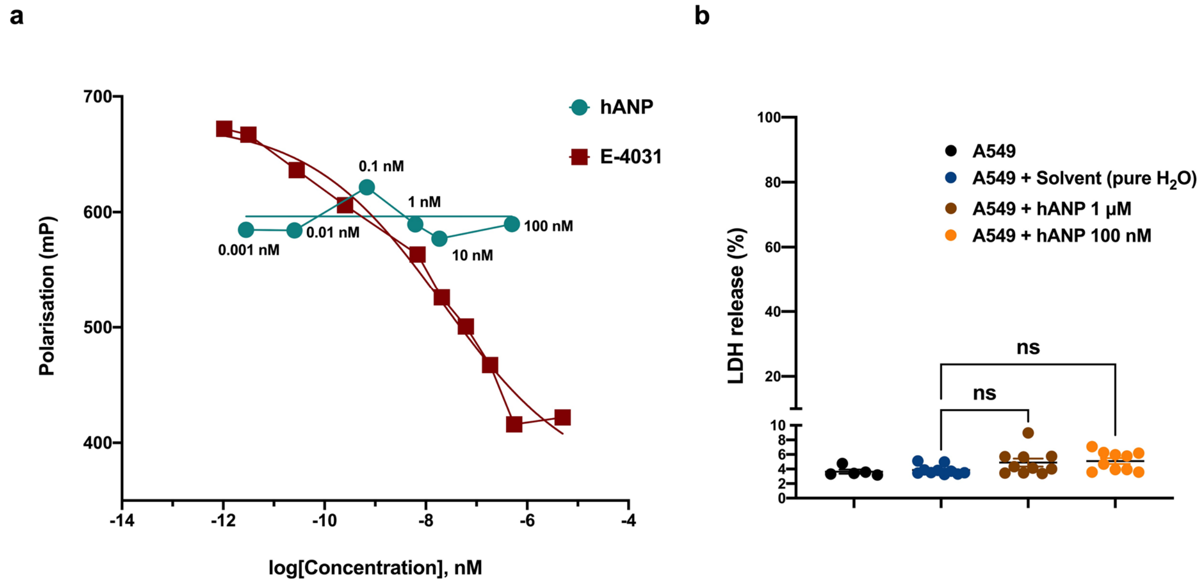

Fig. 8. a, Concentration response curves for hANP and E-4031. hANP binding to the human Ether-a-go-go Related Gene (hERG) channel was assessed using fluorescence polarization (FP). The binding of hANP (green circles) to hERG channel was determined at various concentrations (100 nM to 0.001 nM). E-4031 (red squares) is a compound that is known to bind to the hERG channel, and was used as a positive control molecule. The FP values were recorded using the Spark 20M multimode Microplate Reader. FP values were plotted against compound concentration. The high FP values evoke no binding of the ligand (hANP) to hERG channel, whereas a decrease of FP values in a dose-dependent manner reveal a displacement by an hERG binding molecule (E-4031). **b, Impact of hANP on A549 lung cells.** Percentage of lactate dehydrogenase (LDH) released by A549 lung cells after 18 h exposure to H_2O (solvent for hANP), hANP (1 µM) or hANP (0.1 µM). Data are the result of the analysis of three independent biological assays (n=3) with four or two replicates each. Statistics were achieved by ordinary one-way ANOVA followed by Dunnett's multiple-comparison test. ^{ns}, not significant ($P \geq 0.05$).

Supplementary Tables 1-2

Supplementary Table 1. Bacterial strains and plasmids used in this study

Strain or plasmid	Genotype or relevant characteristics	Reference or source
***Pseudomonas aeruginosa* strains**		
H103	Wild-type; a prototroph derivative of PAO1	Hancock and Carey, 1979
PA14	Wild-type	Rahme *et al*. 1995
PAK	Wild-type	Takeya and Amako. 1966
CF 8.19	Clinical strain Cystic Fibrosis MDR	CNR résistance aux antibiotiques CHU Besançon, France
CF 9.19	Clinical strain Cystic Fibrosis MDR	CNR résistance aux antibiotiques CHU Besançon, France
PAL 0.1	Clinical strain	Grandjean *et al*. 2018
PAL 1.1	Clinical strain	Grandjean *et al*. 2018
MUC-N1	Clinical strain	Boukerb *et al*. 2020
MUC-N2	Clinical strain	Boukerb *et al*. 2020
MUC-P4	Clinical strain	Boukerb *et al*. 2020
MUC-P5	Clinical strain	Boukerb *et al*. 2020
ΔamiC	PA14-*ΔamiC, ID 36129*	Liberati *et al*.2006
ΔamiC Comp	PA14-*ΔamiC* harboring pBBR-MCS4::*amiC*; Cb^R	Rosay *et al*., 2015
ΔamiR	PA14-*ΔamiR*, deletion mutant	This study
AmiR+	PA14 harboring pBBR-MCS4:*amiR*; Cb^R	This study
PA14-EV	PA14 harboring pBBR-MCS4; Cb^R	This study
***Escherichia coli* strains**		
TOP10	Competent cells used as cloning host	Invitrogen
S17.1	*recA pro* (RP4-2Tet::Mu Kan::Tn7), donor and helper strain for conjugation	Simon *et al*., 1983
**Plasmids**		
pBBR-MCS4	Cloning vector; Cb^R	Kovach *et al*, 1995
pBBR-MCS4::*amiC*	pBBR-MCS4 containing intact *amiC*; Cb^R	Rosay *et al*., 2015
pBBR-MCS4::*amiR*	pBBR-MCS4 containing intact *amiR*; Cb^R	This study

pGEM-T	High-copy-number cloning vector, *bla, lacZ*; Ap^R	Promega
pGEM-T::*amiR*	pGEM-T containing 5' and 3' flanking sequence of *amiR* gene; Ap^R	This study
pEX100Tlink	pUC19-based gene replacement vector with MCS[a], *sacB*; Ap^R	Quénée *et al*., 2005
pEXT100Tlink::*amiR*	pEXT100Tlink containing 5' and 3' flanking sequence of *amiR* gene; Ap^R	This study

[a], multiple cloning site
^{R}, antibiotic resistance
E. coli strains were grown in the presence of ampicillin (Ap; 100 μg/mL). *P. aeruginosa* strains were grown in LB liquid medium in the presence of carbenicillin (Cb; 300 μg/mL) or on LB agar medium containing Cb (600 μg/mL).

Supplementary Table 2. List of primers used in this study

Primer name	Sequence (5'-3')[a]	Lenght
Construction of the *P. aeruginosa* PA14-Δ*amiR* deletion mutant strain		
*amiR*_1-*SacI*-F	taataa-***gagctc***-CATCGGCTCGGACTACATCTATCCG	37
*amiR*_1-R	TCAGGCGGAGGCGCTCATGGGAGCGCT	27
*amiR*_2-F	ATGAGCGCCTCCGCCTGAGCGATACCGG	28
*amiR*_2-*HindIII*-R	taataa-***aagctt***-GGAAACCTTGTCGTTCGGTTTCGG	36
Construction of the *P. aeruginosa* PA14-AmiR+ overexpressing strain		
AmiR-*XhoI*-F	taataa-***ctcgag***-GCATCGCGGAAATCGATG	30
AmiR-*XbaI*-R	taataa-***tctaga***-ACAGGCCGGTATCGCTCA	30

[a], All the primers used in this study were synthesized by Eurogentec and are based on *P. aeruginosa* UCBPP-PA14 genome sequence (http://www.pseudomonas.com). Bold underlined nucleotides indicate restriction endonuclease sites inserted within primer sequences.

Supplementary Methods

Caenorhabditis elegans virulence assay

The *Caenorhabditis elegans* wild-type Bristol strain N2 was obtained from the *Caenorhabditis* Genetics Center (Minneapolis, MN, USA) which is funded by NIH Office of Research Infrastructure Programs (P40 OD010440). *C. elegans* were maintained under standard culturing conditions at 22°C on nematode growth medium agar plates with *Escherichia coli* OP50 as a food source (Sulston and Hodgkin, 1988). Synchronous worm cultures were generated as previously described (Blier et al. 2011). Bacterial virulence was evaluated using the liquid killing assay as previously described with slight modifications (Chua et al., 2014). Briefly, from an overnight bacterial growth, *P. aeruginosa* PA14 was inoculated at $OD_{580\ nm}$ = 0.08 into LB supplemented with hANP as appropriate. After two hours, bacteria were collected by centrifugation (2 min at 8 000 *g*), cells were resuspended into CELKA medium to OD_{580nm} = 0.1 and used for virulence assay on *C. elegans* (Chua et al., 2014). Twenty to twenty-five L4 (48 hours old) synchronized worms, harvested with M9 solution, were added to 80 µL of *P. aeruginosa* PA14 previously exposed to hANP (at different concentrations) and non-exposed controls and incubated at 22°C. Worm survival was scored at 1 hour and each following hour using an Axiovert S100 optical microscope (Zeiss, Oberkochen, Germany). The worms were considered dead when they remained static without grinder movements for 20 s or did not respond to light flashes. Results are expressed as a percentage of living worms. The results are the average of three independent assays. For the *C. elegans* killing assay, nematode survival was calculated by the Kaplan-Meier method (GraphPad Prism version 9.0; GraphPad Software, San Diego, California, USA).

Hydrogen cyanide assay

Hydrogen cyanide concentration in *P. aeruginosa* PA14 cultures, unexposed (control condition) or exposed to various concentrations of hANP, was determined by the polarographic technique described by Blier *et al.* (2012). Bacterial cultures were centrifuged 10 min at 8000 *g* and the supernatant was passed through a 0.22 µm filter to remove all bacterial bodies. The filtered supernatant was then diluted in 0.2 mol.L^{-1} borate electrolyte (pH=10.2). The solution was purged with N_2 for 3 min to remove dissolved oxygen and then for 20 s more between each addition of cyanide potassium (KCN) that was used as an internal standard. A scan of the electric potential was carried out in the cathodic negative sense from -0.1 V to -0.5 V with a sweep rate of 10 mV/s. The pulse amplitude was 0.05 V with a pulse duration of 0.04 s. The peak height of cyanide was measured at -200 mV in a differential pulse mode and the cyanide concentration was determined by the addition of 4 successive aliquots of 10 mg.L^{-1} KCN standards.

Cell culture

The human A549 lung epithelial cell type II line (ATCC-CCL185TM) (American Type Culture Collection Manassas, VA) was grown at 37°C in 5% CO_2 atmosphere in Dulbecco's modified Eagle's medium (DMEM, Lonza) supplemented with 10% fetal calf serum (Lonza) and 1% (w/v) penicillin and streptomycin (Penistrep, Lonza). Routinely, cells were seeded in a 25 mL flask and used at 80% confluence. For cytotoxicity assays, cells were seeded in 24 well plates at a final density of $3x10^5$ cells per well and grown for 48 h before use. A minimum of 24 h before infection assays, cells were deprived of antibiotics and fetal calf serum by addition of a fresh serum-free medium.

Measurement of the release of cytosolic lactate dehydrogenase (LDH) by A549 cells

Lactate dehydrogenase (LDH) is a stable cytosolic enzyme released into the culture medium after cell lysis. The amount of LDH released by eukaryotic cells in the presence of the bacteria, exposed to various concentration of hANP, was determined using the Cytotox 96 enzymatic assay (Promega, Charbonnières, France). A549 cells were incubated for 6 h with control or pre-treated *P. aeruginosa* PA14 at a multiplicity of infection of 10. A lysis buffer, consisting of a solution of Triton X-100 (9 % (v/v) in water), was employed to determine the maximum LDH release by A549 cells in our experimental conditions (100 % LDH release). A background level was established using culture medium alone, and defined as 0 % LDH release, to eliminate the contribution of the culture medium. The percentage of LDH release in the cell population was then calculated using the equation:

$$\%\text{LDH} = \frac{\text{OD sample}}{\text{OD } 100\%} \text{ X } 100$$

The assay was sufficiently sensitive to measure a concentration of LDH equivalent to the lysis of 1 % of the cell population.

Determination of hANP binding on human ether-a-go-go (hERG) channel

Affinity of hANP for the hERG channel was determined using the Predictor™ hERG fluorescence polarization (FP) assay kit (Thermo Scientific) following the manufacturer's instructions. Briefly, when the red-shifted fluorescent tracer is bound to the hERG channel, the FP value produced is high. If tested compounds can displace the tracer interacting with the hERG channel, the FP value is low. The hANP assayed concentrations ranged between 100 nM (the highest concentration) to 0.001 nM. The E-4031 molecule used as a positive control was diluted from the stock solution (15 µM) to a concentration of 10^{-14} M. The plate was read using

the Spark 20M multimode Microplate Reader (Tecan Group Ltd., Männedorf, Switzerland), with the excitation and emission wavelengths set to 535 nm (25 nm bandwidth) and 595 nm (35 nm bandwidth), respectively.

Membrane fluidity

Fluorescence anisotropy analysis of *P. aeruginosa* PA14 unexposed (control condition) or exposed to hANP was performed as described by Vincent et al. (Vincent et al., 2004) with a few modifications. *P. aeruginosa* PA14 strain was grown in 10 mL LB broth at 37°C under agitation (180 rpm). After 2 h growth, the bacterial cultures were untreated (control condition) or hANP-treated at the required concentration. The bacterial cultures were allowed for an additional 3 h incubation. Bacterial cells were then harvested by centrifugation (5 min, 7500 *g*), washed twice in 10 mM $MgSO_4$ and resuspended in the same wash solution to an OD_{580} of 0.1. Next, 1 µL of a 4 mM stock solution of 1,6-diphenyl-1,3,5-hexatriene (DPH) (Sigma-Aldrich) in tetrahydrofuran was added to a 1 mL aliquot of the resuspended cultures and incubated in the dark for 30 min at 37°C to allow the probe to incorporate into the membrane bilayer. Measurement of the fluorescence polarization was performed using the Spark 20M multimode Microplate Reader (Tecan) equipped with the Te-Cool™ active temperature regulation system. Excitation and emission wavelengths were set to 365 and 425 nm, respectively, and the anisotropy *(r)* was calculated according to Lakowicz (Lakowicz, 2006). Data were recorded using SparkControl™ software (Version 2.1, Tecan). Fluorescence polarization and membrane fluidity are inversely related since increasing anisotropy values correspond to a less fluid membrane environment and vice versa. All values reported for each sample are means of eight measurements of at least three independent experiments.

Supplementary References

Blier, A. S. *et al.* C-type natriuretic peptide modulates quorum sensing molecule and toxin production in *Pseudomonas aeruginosa*. *Microbiology (Reading)* **157**, 1929-1944, doi:10.1099/mic.0.046755-0 (2011).

Blier, A. S. *et al.* Quantification of *Pseudomonas aeruginosa* hydrogen cyanide production by a polarographic approach. *J Microbiol Methods* **90,** 20–4, doi: 10.1016/j.mimet.2012.04.005 (2012).

Boukerb, A. *et al.* Draft Genome Sequences of Four *Pseudomonas aeruginosa* Clinical Strains with Various Biofilm Phenotypes. *Microbiol Resour Announc*., **9(1)**:e01286-19, doi: 10.1128/MRA.01286-19 (2020).

Chua, S. L. *et al.* Dispersed cells represent a distinct stage in the transition from bacterial biofilm to planktonic lifestyles. *Nat Commun* **5**, 4462, doi:10.1038/ncomms5462 (2014).

Grandjean, T., Le Guern, R., Duployez, C., Faure, K., Kipnis, E., Dessein, R. Draft Genome Sequences of Two *Pseudomonas aeruginosa* Multidrug-Resistant Clinical Isolates, PAL0.1 and PAL1.1 *Microbiol Resour Announc*.; **7(17)**:e00940-18, doi: 10.1128/MRA.00940-18. eCollection (2018).

Hancock, R. E. W. & Carey, A. M. Outer membrane of *Pseudomonas aeruginosa*: Heat- and 2-mercaptoethanol-modifiable proteins. *J. Bacteriol.* **140**, 902–910, doi: 10.1128/jb.140.3.902-910.1979 (1979).

Kovach, M. E., Phillips, R. W., Elzer, P. H., Roop, R. M., 2nd & Peterson, K. M. pBBR1MCS: a broad-host-range cloning vector. *Biotechniques* **16**, 800-802 (1994).

Lakowicz, J.R. Principles of Fluorescence Spectroscopy, 3rd ed.; Springer: Boston, MA, USA; ISBN 978-0-387-31278-1 (2006).

Laskowski RA, Swindells MB. LigPlot+: multiple ligand-protein interaction diagrams for drug discovery. *J Chem Inf Model*.;**51**(10):2778-86. doi: 10.1021/ci200227u (2011).

Liberati, N. T. *et al.* An ordered, nonredundant library of *Pseudomonas aeruginosa* strain PA14 transposon insertion mutants. *Proc Natl Acad Sci U S A* **103**, 2833-2838, doi:10.1073/pnas.0511100103 (2006).

Quénée, L., Lamotte, D. & Polack, B. Combined *sacB*-based negative selection and *cre-lox* antibiotic marker recycling for efficient gene deletion in *Pseudomonas aeruginosa. BioTechniques* **38**, 63–67, doi: 10.2144/05381ST01 (2005).

Rahme LG, Stevens EJ, Wolfort SF, Shao J, Tompkins RG, Ausubel FM. Common virulence factors for bacterial pathogenicity in plants and animals. *Science*.;**268**(5219):1899-902. doi: 10.1126/science.7604262 (1995).

Rosay, T. *et al. Pseudomonas aeruginosa* expresses a functional human natriuretic peptide receptor ortholog: involvement in biofilm formation. *mBio* **6**, doi:10.1128/mBio.01033-15 (2015).

Simon, R., Priefer, U. & Pühler, A. A broad host range mobilization system for in vivo genetic engineering: Transposon mutagenesis in Gram negative bacteria. *Bio/Technology* **1**, 784–791 (1983).

Sulston, J. & Hodgkin, J. *The Nematode* Caenorhabditis elegans. (Wood, W. B., 1988).

Takeya K, Amako K., A rod-shaped *Pseudomonas* phage. *Virology* 28:163–165. doi:10.1016/0042-6822(66)90317-5 (1966).

Vincent, M.; England, L.S.; Trevors, J.T. Cytoplasmic membrane polarization in Gram-positive and Gram-negative bacteria grown in the absence and presence of tetracycline. *Biochim. Biophys. Acta*, **1672**, 131–134, doi:10.1016/j.bbagen.2004.03.005, (2004).
